# Quorum sensing and DNA methylation play active roles in clinical *Burkholderia* phase variation

**DOI:** 10.1128/jb.00531-24

**Published:** 2025-02-14

**Authors:** Pauline M. L. Coulon, Marie-Christine Groleau, Abderrahman Hachani, Matthew P. Padula, Timothy P. Stinear, Eric Déziel

**Affiliations:** 1 Australian Institute for Microbiology and Infection, Faculty of Science, University of Technology Sydney 1994 , Ultimo, New South Wales, Australia; 2 Centre Armand-Frappier Santé Biotechnologie, Institut National de la Recherche Scientifique 14851 , Laval, Québec, Canada; 3 Department of Microbiology and Immunology, Doherty Institute, University of Melbourne 198084 , Parkville, Victoria, Australia; 4 School of Life Sciences, Faculty of Science, University of Technology Sydney 1994 , Ultimo, New South Wales, Australia; 5 Centre for Pathogen Genomics, University of Melbourne 2281 , Melbourne, Victoria, Australia; University of Illinois Chicago, Chicago, Illinois, USA

**Keywords:** bacterial communication, epigenetics, colony morphotype, virulence

## Abstract

Phenotypic diversity in bacteria often results from adaptation to changing environmental conditions, exemplified by variable colony morphotypes. In *Burkholderia pseudomallei*, discrete genomic alterations and modulation of gene expression facilitate adaptation. Adapted variants of species within the *Burkholderia cepacia* complex (Bcc) often lose the pC3 virulence megaplasmid, impacting their colony morphology and their production of virulence factors. In this study, we characterize variants arising in *Burkholderia ambifaria* clinical isolates using proteomics and phenotypic tests and show that some of them have retained the pC3, indicating a distinct phase variation mechanism at play in this Bcc species. Interestingly, variants of *B. ambifaria* strains CEP0996 (pC3-null) and HSJ1 (pC3-positive) still share similarities in phenotypes controlled by the Cep quorum-sensing (QS) system. We further investigated the role of QS in *B. ambifaria* HSJ1 phase variation and confirmed that the Cep QS system is important for the emergence of variants. Given that DNA methylation is a key epigenetic factor regulating virulence factors in *Burkholderia cenocepacia*, we hypothesized that adenosine DNA methylation also governs phase variation in *B. ambifaria* HSJ1. By deleting the genes encoding putative adenosine DNA methyltransferases, we discovered that an orphan type II DNA methyltransferase inhibits the emergence of phase variants. This study is the first to demonstrate that quorum sensing and adenosine DNA methylation are two antagonistic systems independently controlling phase variation in *B. ambifaria*.

**IMPORTANCE:**

Some *Burkholderia* species are pathogenic to plants, animals, or humans. In immunocompromised individuals, and people suffering from cystic fibrosis, infection from the *Burkholderia cepacia* complex (Bcc) can lead to “*cepacia* syndrome.” In northern Australia and southeast Asia, melioidosis caused by *Burkholderia pseudomallei* is prevalent among native population, particularly among people with diabetes, chronic lung or kidney disease or alcoholism. *Burkholderia*’s phenotypic plasticity, including colony morphotype variation (CMV), enables rapid adaptation to diverse environments, enhancing its survival and pathogenicity. This study reveals phase variation as a new CMV mechanism within the Bcc group and is the first to report that quorum sensing and DNA methylation are involved in phase variation. Understanding the underlying mechanisms of CMV could lead to the development of targeted therapies against these highly antibiotic-tolerant bacteria.

## INTRODUCTION

The *Burkholderia* genus encompasses both plant-beneficial and pathogenic species that are broadly distributed in various environments, such as soils, water, or plant rhizosphere (1, 2). For instance, *Burkholderia glumae* and *Burkholderia gladioli* are well-described plant pathogens associated with bacterial panicle blight or leaf strike (3–6). Among mammalian opportunist pathogens, the *Burkholderia pseudomallei* complex (Bpc) includes *Burkholderia pseudomallei* (*Bp*) and *Burkholderia mallei*, causing melioidosis in humans and glanders in animals, respectively (7–9). In contrast, although some members of the *Burkholderia cepacia* complex (Bcc) were once valued for their bioremediation and plant growth-promoting properties (10–14), others have emerged as a significant concern due to their association with severe pneumonia and the “cepacia syndrome” in people with cystic fibrosis (CF) and other immunocompromised patients (1, 15–17). Understanding the mechanisms behind *Burkholderia* virulence and persistence during infection is essential, especially given their intrinsic tolerance and resistance to many clinically relevant antibiotics (18–20).

Colony morphotype variation (CMV) is a process that enables bacteria to adapt to changing environmental conditions (21, 22). This phenomenon can occur through various mechanisms, including (i) genomic variations (e.g., single nucleotide polymorphism, indels, or sequence inversion) (23), (ii) modulation of gene expression (24–28), or (iii) epigenetic factors such as DNA methylation (21, 29, 30). CMV reflects in phenotypic changes that can enhance bacterial fitness and has been described in *Burkholderia* species (22, 23, 27, 31–38). Among Bcc members, CMV was observed among clinical *Burkholderia ambifaria* (*Ba*) isolates, where variant colonies are distinguishable by their smooth and translucent phenotypes, contrasting with the rough and wrinkled appearance of the parental colony (22).

In our previous work, we described the switch of clinical *Ba* strains to variants displaying environmental-like phenotypes linked to a better adaptation to the rhizosphere. These variants exhibit reduced expression of virulence determinants, such as alginate-like exopolysaccharides (EPS), hemolysin, cholesterol oxidase and antifungal activities, as well as production of 4-hydroxy-3-methyl-2-alkylquinolines (HMAQ) (22). In addition, Bernier et al. (25) reported that shiny variant colonies (shv) appear from the clinical *Burkholderia cenocepacia* (*Bc*) strain K56-2 due to a mutation in the LysR-type transcriptional shiny variant colony regulator (ShvR), which also influences antimicrobial resistance, protease and EPS production, and biofilm formation (25, 26, 39–41).

Creating a Tn*5* random mutagenesis library in the clinical *Bc* strain H111, Agnoli et al. (31) discovered that multiple non-pathogenic shiny colony clones had lost their megaplasmid of virulence (pC3). The curing of the pC3 from six Bcc strains resulted in the attenuation of virulence in infection models alongside reduced antifungal activity, EPS and protease production, and altered substrate utilization (31, 42, 43). Although the loss of pC3 has been described as an adaptation to laboratory conditions, four environmental *Burkholderia ubonensis* (*Bu*) isolates (44) and one clinical *Bc* isolate (36) were reported to be lacking pC3, suggesting that this phenomenon can occur naturally.

Recent studies have revealed that the pC3 megaplasmid is stabilized by DNA methylation in *Bc* strains J2315 and H111, where deletion of all three adenosine DNA methyltransferases increased the frequency of variants lacking pC3 (45). DNA methylation serves as an ON/OFF regulatory mechanism modifying specific motifs in the promoter regions of genes associated with virulence functions, including biofilm formation, cell aggregation, and motility (46).

Most virulence factors in *Burkholderia* are controlled by quorum sensing (QS), an intercellular communication system dependent on cell-cell signaling to synchronize target gene transcription within a bacterial population (47). In members of the Bcc, QS is typically mediated by LuxI/LuxR type systems (48), comprising an acyl-homoserine lactone (AHL) synthase (CepI) and a cognate transcriptional regulator (CepR). The AHLs produced by CepI bind specifically to CepR, forming an activated autoregulatory complex that controls the expression of *cepI*, *cepR,* and several target genes related to virulence (49–53).

Moreover, in *Bc*, QS regulation interacts with other regulatory elements such as the *Burkholderia* diffusible signal factor (BDSF) system, which inhibits cyclic-di-GMP production, thereby affecting biofilm formation (54–58). ShvR, which affects the expression of over one thousand genes—including those co-regulated by QS—plays a crucial role in this complex regulatory network (25, 26, 39–41).

In this study, we report that variants of clinical *Ba* strains (22) actually belong to two distinct groups: (i) pC3-positive phase variants; and (ii) pC3-null variants. This new classification highlights the potential presence of diverse mechanisms underlying CMV in *Burkholderia*, suggesting that further investigations are required to understand CMV’s role in virulence and environmental adaptation.

## RESULTS

### Clinical *B. ambifaria* strains generate two distinct variant types, including one that loses its pC3 replicon

We have previously reported that eight clinical *Ba* isolates can undergo CMV by generating phenotypically distinguishable variants on agar plates containing Congo Red (22). One characteristic of *Ba* variants is the lack of production of secondary metabolites belonging to the HMAQ family; these molecules are produced by enzymes encoded by the *hmqABCDEFG* operon, which is normally located on the pC3 replicon in Bcc members (22, 59, 60). We previously established the prevalence of the Hmq system in the Bcc using PCR primers specific to the first and the last gene of the *hmq* operon (*hmqA* and *hmqG*) (60). Using the same method to investigate our panel of eight clinical *Ba* isolates, we found that both *hmqA and hmqG* genes are absent from two of these eight strains (Table 1). Interestingly, out of the six remaining clinical strains carrying the *hmq* operon, the *hmqA* and *hmqG* genes were not detectable in four of their variants (Table 1). Together, these results hinted at a possible loss of the hmq operon in up to six out of eight *Ba* clinical strains.

**TABLE 1:** A majority of phenotypic variants derived from eight clinical *Ba* isolates have lost their pC3 replicon^*a*^

Strains		*hmqA*	*hmqG*	*cepI2*	BAMB_RS28665	BAMB_RS29710	BAMB_RS31530
*B. ambifaria* CEP0516	Parental	−	−	+	+	+	+
	Variant	−	−	×	×	×	×
*B. ambifaria* CEP0617	Parental	+	+	+	+	+	+
	Variant	×	×	×	×	×	×
*B. ambifaria* CEP0958	Parental	+	+	+	+	+	+
	Variant	×	×	×	×	×	×
*B. ambifaria* CEP0996	Parental	+	+	+	+	+	+
	Variant	×	×	×	×	×	×
*B. ambifaria* AU4157	Parental	+	+	+	+	+	+
	Variant	×	×	×	×	×	×
*B. ambifaria* AU8235	Parental	−	−	+	+	+	+
	Variant	−	−	×	×	×	×
*B. ambifaria* HSJ1	Parental	+	+	+	+	+	+
	Variant	✓	✓	✓	✓	✓	✓
*B. ambifaria* AU0212	Parental	+	+	+	+	+	+
	Variant	✓	✓	✓	✓	✓	✓

^*a*^ +, gene detected**; −,** gene not detected; ×, loss of gene vs parental background; and ✓, gene retained vs parental background.

Given previous reports of *Bcc* isolates losing their pC3 replicon (31, 36, 42–44), we performed a PCR screen for three additional genes located on the pC3 replicon (*cepI2*, BAMB_RS28665, and BAMB_RS31530). Consistent with our initial *hmq* gene screen (60), no amplification was observed in six out of eight tested variants, while amplification was positive for their respective parental strains (Table 1). These results indicate that several *Ba* variants have indeed lost the pC3 replicon and thus are classified as “pC3-null.” As expected, *hisA,* a gene discriminating Bcc species and located on chromosome 1 (61), was used as a positive control and was indeed detected in both the parental and variant strains. In contrast, variants of the two other *Ba* strains HSJ1 and AU0212 were positive for all screened genes, including *hmqA* and *hmqG*, despite not producing HMAQs (Table 1 [22, 60]). These PCR analyses were confirmed by whole-genome sequencing of the parental and variants of strains CEP0996 and HSJ1, representing both types of variants (with or without pC3) (Fig. S1). Mapping each variant’s Illumina reads onto their respective parental strain’s whole-genome assembly confirmed that the CEP0996 variant (CEP0996v) has conserved two chromosomes but has lost pC3 (Fig. S1A), while both chromosomes and pC3 are still present in HSJ1v (Fig. S1B). CEP0996 and its pC3-null variant (CEP0996v, pC3-null) along with HSJ1 and its pC3-positive variant (HSJ1v) were chosen as models to further characterize phase variation in *Ba*.

### Both types of variants share similarities and dissimilarities in the production of QS-regulated factors

Since several phenotypes are in common between both types of variants, such as loss of HMAQ production (22), we sought to further characterize the different features of pC3-null variant CEP0996v compared to pC3-positive variant HSJ1v.

**Fig 1:**
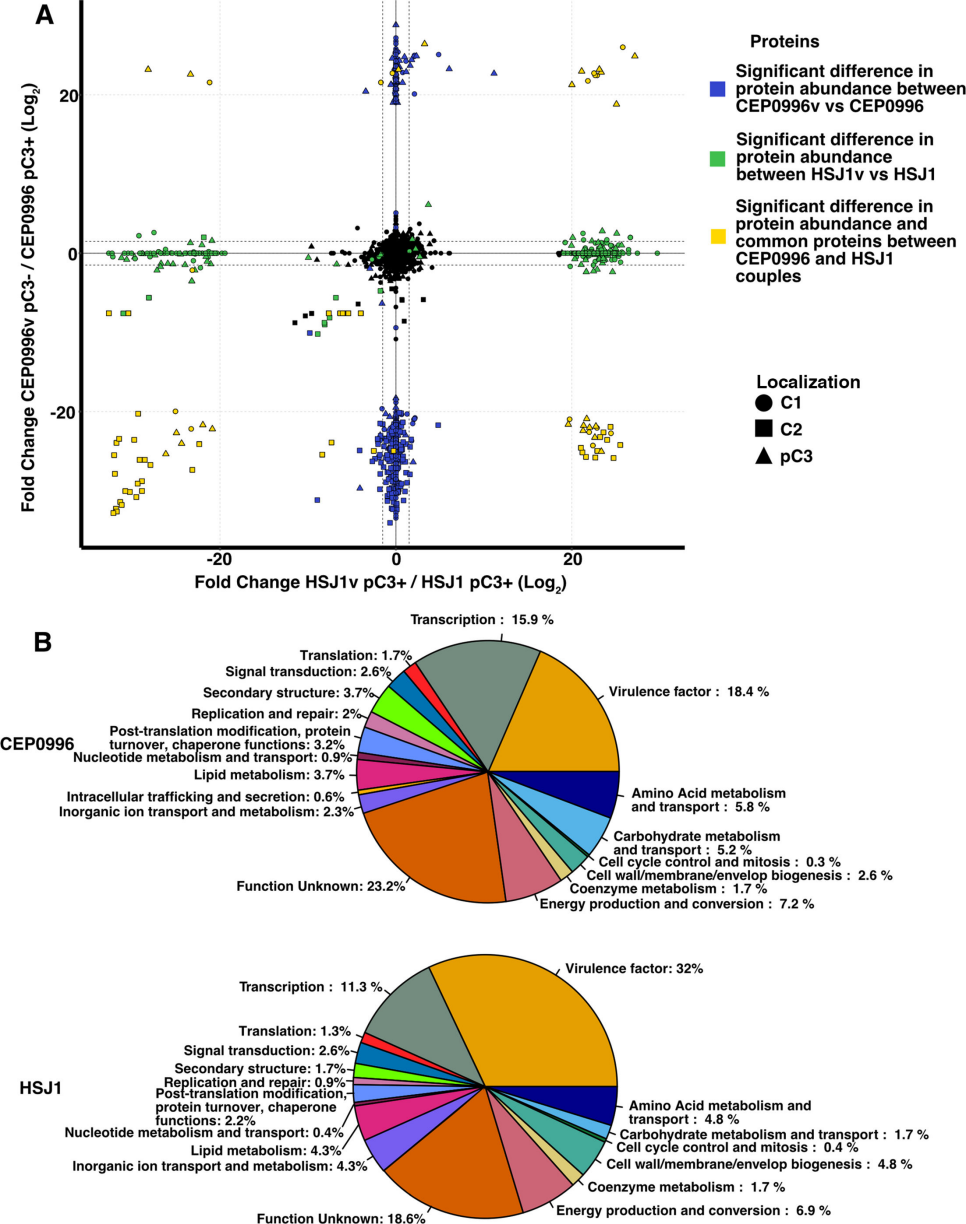
Both types of variants showed dissimilarities in their protein production signatures. (**A**) Proteomes of parental and variants for CEP0996 and HSJ1. Significative differences in protein abundance between CEP0996v and CEP0996 are shown in blue. Significative differences in protein abundance between HSJ1v and HSJ1 are shown in green. Significant common proteins between CEP0996 and HSJ1 couples are shown in yellow. Proteins located on chromosome 1 are represented by circles. Proteins located on chromosome 2 are represented by squares. Proteins located on pC3 are represented by triangles. The abundance was assigned a value of “0” (instead of “NA”) for proteins that were undetectable in one strain but detected in the other to allow their representation on the plot. This conversion enables a clear comparison of protein presence/absence across samples. Dashed lines represent the *P*-value cutoff at 0.05 on a Log2 scale. In the top right quadrant, proteins increased in abundance in both variants, suggesting shared regulatory mechanisms, potentially due to the loss of a repressor or activation of common pathways. Proteins in the top left quadrant are more abundant in CEP0996v but decreased in HSJ1v, indicating differential regulation. The bottom right quadrant represents proteins increased in abundance in HSJ1v but decreased in CEP0996 pC3-null, suggesting activation in phase variation but suppression upon the loss of pC3. Finally, proteins in the bottom left quadrant decreased in abundance in both variants, likely reflecting shared losses in pathways dependent on pC3 or regulatory elements impacted by both phase variation and pC3 absence. (**B**) Significant proteins have been classified by clustering of orthologous groups.

The absence of production of HMAQs suggested that some genes on the pC3 of HSJ1v are present but not expressed when the bacterium is in its variant state. We, therefore, compared the proteomes of both variants with their parent strains when cultured in tryptic soy broth to an OD_600_ of ~1 (Fig. 1). Data-dependent acquisition shotgun proteomic enabled the quantification of 2,872 proteins out of 6,508 open reading frames (ORFs) for CEP0996 and 2,850 proteins out of 6,700 ORFs for HSJ1 across all replicates (Fig. S2). Our analysis identified 396 proteins that were differently detected in CEP0996 pC3-null (variant) compared to its parent. Of these, 104 are encoded on chromosome 1, 112 on chromosome 2, and 180 on the pC3 replicon (Tables S6 and S7). For HSJ1, 328 proteins were differently detected in the variant vs the parental strain, plus 20 detectable proteins whose abundance differed significantly (with an FDR-adjusted *P*-value below 0.01) between HSJ1 and HSJ1v. Of these, 122 are encoded on chromosome 1, 125 on chromosome 2, and 101 on pC3 (Tables S8 and S9).

Among the 6,461 corresponding homologous ORFs between CEP0996 and HSJ1 (Table S10), 77 exhibited significant differences in abundance in variants compared to their parent, including 16, 28, and 32 proteins encoded on chromosomes 1, 2, and pC3, respectively, along with one protein encoded on CEP0996 chromosome 1 but located on chromosome 2 in HSJ1 (Table S11). Fold changes in protein abundance between HSJ1v and CEP0996v (pC3-null) were further analyzed and categorized into four quadrants based on expression patterns (Fig. 1A). Proteins in each quadrant reflect shared regulatory mechanisms or differential regulation influenced by phase variation and the presence or absence of pC3, highlighting common pathways, specific activations, or suppressions linked to these conditions. Overall, most of the proteins showing difference in abundance between each *Ba* parental isolate and its respective variant are linked to primary metabolism and energy production, antibiotic biosynthesis clusters, and virulence factors (Fig. 1B; Tables S10 and S11). Notably, proteins involved in the biosynthesis of type II–IV pilus, cepacian EPS, and various secondary metabolites with known antimicrobial properties (including AFC lipopeptide, enacyloxin, HMAQs, occidiofungin, and pyrrolnitrin) (62–68) exhibited reduced abundance in both types of variants compared to their parents (Table S10). In contrast, proteins linked to virulence determinants—such as adhesins and siderophores, increased in abundance in the variants, while proteins involved in flagellum biosynthesis were in higher abundance in CEP0996v and lower abundance in HSJ1v (Table S10).

### Phenotypic comparison between parental strains and variants

**Fig 2:**
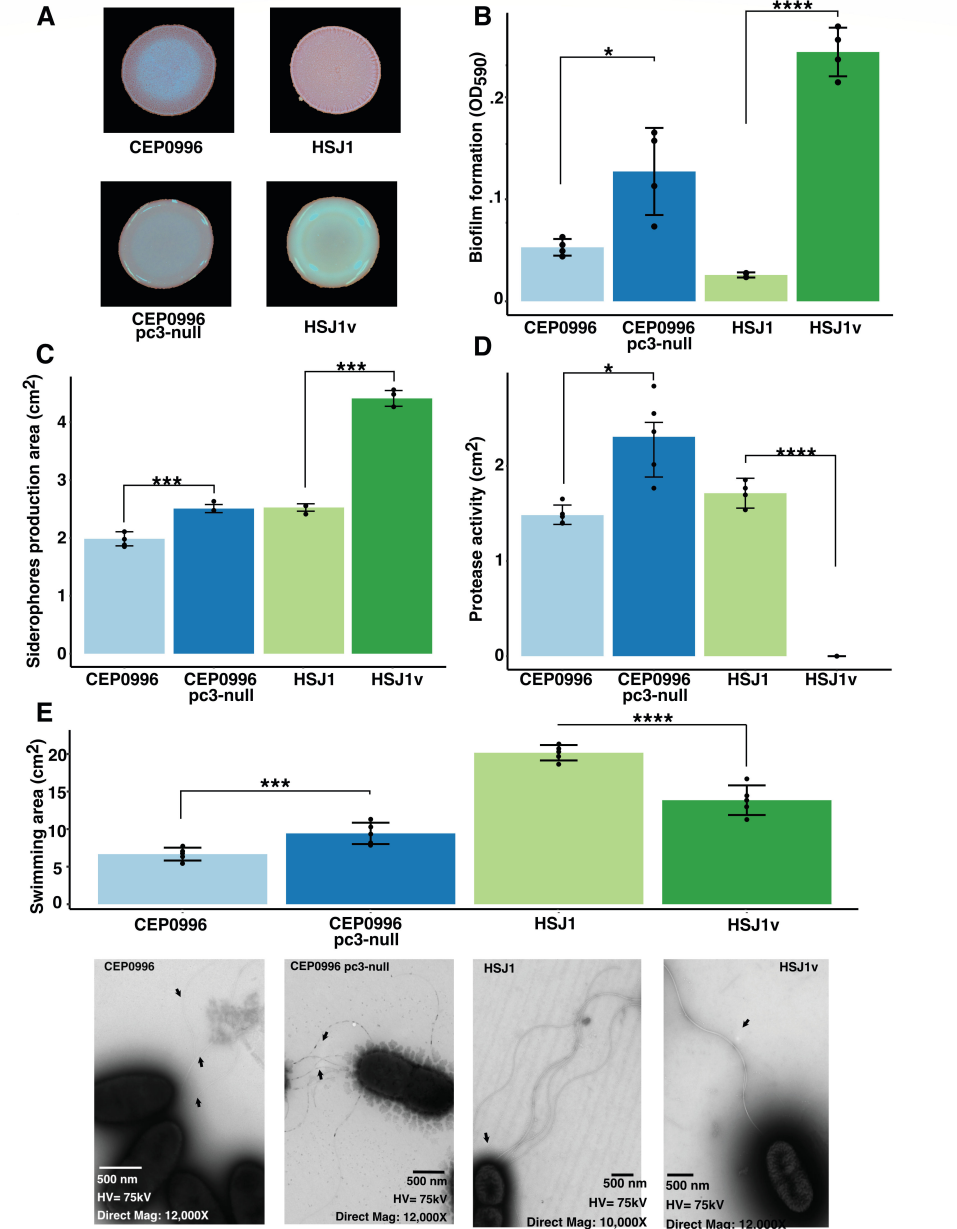
Phenotypic assays in both types of variants compared to their respective parental strain. (**A**) Colony morphologies for each morphotype on CRTSA. (**B**) Biofilm production (Wilcoxon test). (**C**) Siderophore production (Wilcoxon rank test). (**D**) Protease production (*t*-test). (**E**) Swimming motility (*t*-test) coupled with TEM microscope of bacterium flagella. *P*-values are represented by * between 0.5 and 0.01, ***0.001 and 0.0001, and ****inferior to 0.0001. Mean ± SD are shown in each figure.

Next, we assessed phenotypes between parental strains and their corresponding variants, including colony morphologies on Congo Red plates, biofilm production, swimming motility, flagella observation using electron microscopy, and siderophore production (22, 31) (Fig. 2; Table S12). In bacteria, Congo Red binds to different EPS such as cellulose or amyloid fibers (69, 70). While neither strain encoded homologous genes for amyloid adhesins, our proteomic analysis revealed that proteins encoded by the bacterial cellulose synthase (*bcs*) cluster genes are similarly produced by parent and variants (71) (Tables S6 and S8). Thus, the difference in Congo Red binding by both variant colonies (Fig. 2A) could be explained either by a lack of EPS cepacian production on Congo Red agar (72), by a difference in other EPS production (73), such as galactan-3-deoxy-D-manno-oct-2-ulosonic acid (74), or by a change in other putative capsular polysaccharide biosynthesis proteins (75). Variants derived from clinical *Ba* isolates produce more EPS resulting in a more mucoid appearance of the colony (22, 72), correlating with the enhanced biofilm-forming ability of CEP0996v and HSJ1v compared to their parental strains (73, 74) (Fig. 2A and B). Both types of variants also produced more proteins associated with siderophore biosynthesis (Fig. 2C), leading to correlated increases in Congo Red binding, biofilm, and siderophore production. Notably, CEP0996 pC3-null has higher proteolytic activity than the parental strain (Fig. 2D), which may be attributed to the production of the protease ZmpA (Tables S7, S9, and S11). Conversely, HSJ1v lacks protease activity, likely due to a decreased abundance of ZmpA and ZmpB (Fig. 2D; Table S9).

In Bcc, flagellar motility is linked to host cell invasion and immune system activation via TLR5; thus, reduced motility is described during persistence, although motility may persist during chronic infections (75–78). Interestingly, proteins involved in flagella were present in higher abundance in CEP0996v compared to its parental strain (Table S10), correlating with increased swimming motility (Fig. 2E). In contrast, HSJ1v showed elevated level of FlgM—an anti-sigma factor that inhibits the transcription of flagellar genes (79)—with both MotA (BAMB_HSJ1_RS_00485) and FliC (BAMB_HSJ1_RS_00535) present in lower abundance (Table S10). This is consistent with a reduced number of flagella, based on our qualitative data from microscopy imaging, and diminished swimming phenotype of HSJ1v compared to its parent (Fig. 2E).

### The *B. ambifaria* HSJ1v phenotypes are not due to genomic changes

For the CEP0996v variant, the observed impact on the expression of several phenotypes is explained by the loss of the pC3 replicon (31). In contrast, since HSJ1v retains its pC3, we expected that the numerous phenotypic differences between parent and variant would be linked to genomic variations (21). However, upon mapping Illumina reads of HSJ1v to the HSJ1 assembly, we observed no significant genomic modification (Table S13). Furthermore, although HSJ1 contains inverted-repeat sequences, no inversions were detected between HSJ1 parent and variant genomic sequences (Table S14). Given that a previous study identified a duplicated region as responsible for colony morphotype variants in *Burkholderia thailandensis* (80), we also investigated this hypothesis but found no duplicated regions in *Ba* HSJ1v.

Altogether, these results suggest that phase variation in HSJ1 is likely due to differences in gene expression modulation or epigenetic factors rather than genomic alterations.

### The *Cep* quorum-sensing system impacts phase variation in HSJ1

Given the lack of genomic changes to explain the phenotypic and proteomic differences between HSJ1 and HSJ1v, we hypothesized that QS could underlie these variations since it regulates biofilm formation, siderophore and protease production, and virulence (52, 53). Additionally, *Ba* possesses the BDSF QS system, which modulates c-di-GMP levels and influences biofilm formation in *Bc* (54–58). In our proteomic study of HSJ1 at OD_600_ ~ 1, proteins CepI and CepR were detected, as well as RpfF and RpfR belonging to the BDSF QS system; however, CepI2 was detected while CepR2 was not (Table 2; Table S9).

**TABLE 2:** Quorum sensing-related genes/proteins detected in the proteomic study of strains HSJ1^*a*^

	Locustag	Detection in proteomic analysis	Quantifiable in parental	Quantifiable in variant
*cepI*	BAMB_HSJ1_RS20835	+	2.82 × 10^8^ ± 1.05 × 10^7^	1.47 × 10^8^ ±9.02 × 10^6^
*rsaM*	BAMB_HSJ1_RS20840	+	1.35 × 10^8^ ± 2.80 × 10^7^	1.2 × 10^8^ ± 3.29 × 10^7^
*cepR*	BAMB_HSJ1_RS20845	+	2.27 × 10^7^ ± 1.15 × 10^7^	3.62 × 10^7^ ± 7.57 × 10^6^
*cepI2*	BAMB_HSJ1_RS28550	+	5.62 × 10^7^ ± 1.47 × 10^7^	2.01 × 10^7^ ± 1.59 × 10^6^
*cepR2*	BAMB_HSJ1_RS28485	−	NA	
*rpfF*	BAMB_HSJ1_RS26685	+	5.10 × 10^7^ ± 1.68 × 10^7^	3.46 × 10^7^ ± 1.10 × 10^7^
*rpfR*	BAMB_HSJ1_RS26690	+	2.81 × 10^8^ ± 1.14 × 10^8^	3.61 × 10^8^ ± 4.65 × 10^7^
*shvR*	BAMB_HSJ1_RS29045	+	5.19 × 10^8^ ± 2.00 × 10^8^	0 ± 0

^*a*^ Values based on label-free quantification intensities, after filtering and imputing missing values.

**Fig 3:**
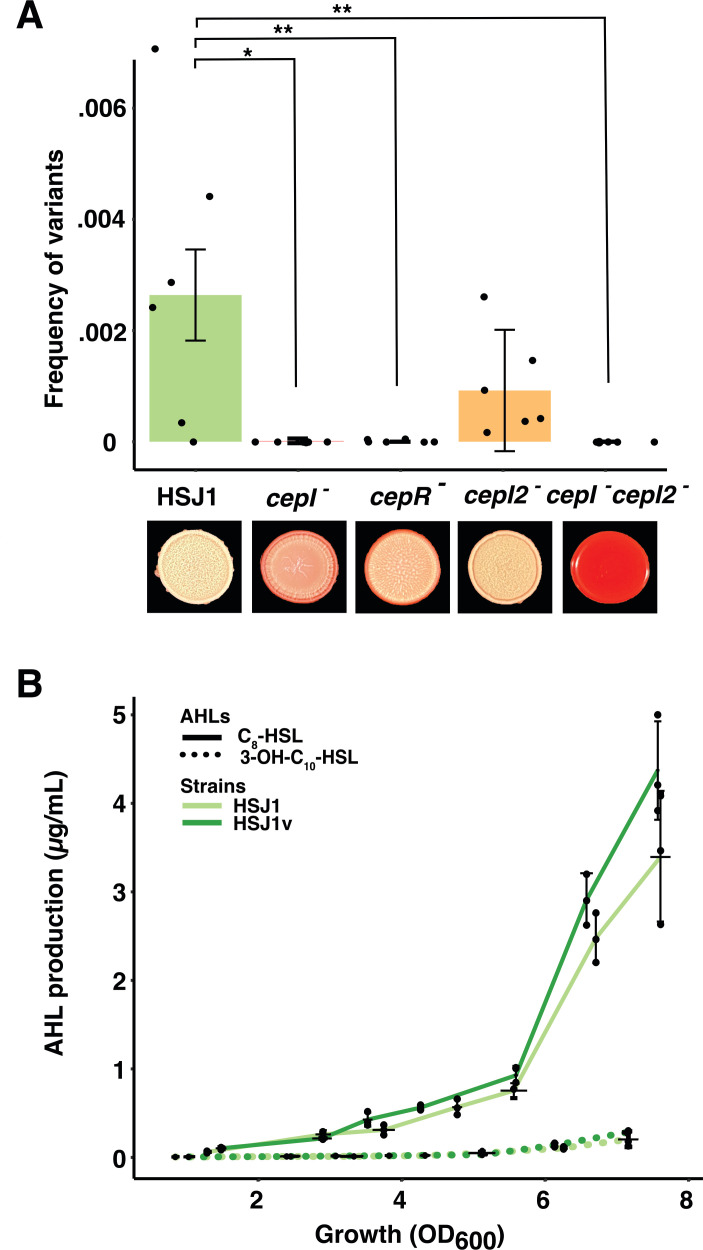
QS activates the production of phase variants in *Ba* HSJ1. (**A**) Frequency of occurrence of variants in QS mutants (Wilcoxon rank test). (**B**) Production of AHLs in HSJ1 parental and variant (ANOVA). *P*-values are represented by * between 0.5 and 0.01 and ** between 0.01 and 0.001. Mean ± SD are shown in each figure.

To assess the direct impact of QS on phase variation, we determined the frequency of variant occurrence in HSJ1 and its isogenic mutants: *cepI^-^, cepR^-^, cepI2^-^,* and *cepI^-^cepI2^-^*. Interestingly, the *cepI^-^, cepR^-^,* and *cepI^-^cepI2^-^* did not produce any variants, while *cepI2^-^* produced variants at the same frequency as the parental strain (Fig. 3A; Table S15). When complemented with a plasmid constitutively expressing the *cepR* gene, the *cepR*^-^ mutant partially regained its ability to generate variants (Fig. S3), confirming that the CepI/CepR QS system is involved in the occurrence of phenotypic variants.

We measured no difference in the production of either AHL QS signal between HSJ1 and its variant (Fig. 3B), in agreement with our previous observation of similar C_8_-HSL production at OD_600_ ~ 4 in both HSJ1 parental and variant (22). It is also the case for CEP0996 and CEP0996v, which produce similar levels of C_8_-HSL. Of note, CEP0996v produces no 3-OH-C_10_-HSL because *cepI2* is located on the missing pC3 (Fig. S4). These results suggest that although QS is important for variant emergence, phase variation does not affect AHL-dependent QS function.

Next, we hypothesized that ShvR, which significantly influences QS, colony morphology, virulence, and biofilm formation in *Bc* strain K56-2, could have a similar function in HSJ1 (26, 41). Interestingly, the protein product of the BAMB_HSJ1_RS29045 gene, which shares 90% similarity at protein level with *shvR* of *Bc* (Fig. S5), is not detected in HSJ1v compared to HSJ1 (Table 2; Table S9), implying that the absence of ShvR is involved in the occurrence and/or phenotypes of HSJ1v. However, the deletion of *shvR* in HSJ1 did not significantly affect the occurrence of variants, colony morphology, or biofilm formation (Fig. S6; Table S16). These data strongly suggest that this protein has a different function in HSJ1 than in *Bc* K56-2.

Overall, these results indicate that the CepI/CepR QS system is important for the emergence of phase variants in HSJ1, while ShvR does not appear to play a significant role.

### DNA methylation inhibits the emergence of phase variants

Since genomic modification or QS cannot explain the significant phenotypic differences observed between HSJ1 and HSJ1v, we hypothesized that DNA methylation could influence phase variation, as it affects similar phenotypes in *Bc* (46). In fact, DNA methylation is an epigenetic modification that can drive phase variation in several bacteria, including *Neisseria* and *Helicobacter* (81).

Using PacBio tools to identify DNA modifications from PacBio sequencing, we identified three adenosine methylation motifs in both strain HSJ1. Since this analysis was conducted on DNA extracted from a single biological replicate cultured overnight, we limit our conclusions to qualitative observations. The identified motifs were identical to those previously reported in the *Ba* AMMD strain (45). Based on these motifs, we assigned the corresponding genes encoding the identified adenosine DNA methyltransferases (MTases) by homology (Table 3). When comparing the three DNA MTase sequences from HSJ1v to its parent, we found no genetic alteration that could suggest differences in expression or function (Fig. S7).

**TABLE 3:** DNA methylation in strain HSJ1^*a*^

Motif	Methylation	Strand	Strain	No. of motif detected^*b*^	No. of motif in genome^*c*^	% motifs detected^*d*^	Mean modif. QV^*e*^	Mean motif coverage	Putative genes and their putative homologs in other Bcc
CAC**A**G	m6A	F	HSJ1	6,265	6,327	96.12	70.03	39.72	BAMB_HSJ1_RS01220-type III (**DNA Mtase 1**)
			HSJ1v	6,260	6,327	98.94	72.94	41.87	
GTWWAC	m6A	F + R	HSJ1	1,578	1,680	93.92	62.86	39.61	BAMB_HSJ1_RS24645-type II orphan (**DNA Mtase 2**)
			HSJ1v	1,599	1,680	95.17	65.13	41.7	
RGATCY	m6A	F + R	HSJ1	11,402	11,862	96.12	68.34	39.76	BAMB_HSJ1_RS09500-type II (**DNA Mtase 3**)
			HSJ1v	11,501	11,862	96.95	69.83	41.87	

^*a*^ The analysis was set up with a minimal *P* value of 0.001, which corresponds to a modification quality values (ModQV) > 30.

^*b*^ No. of motifs detected: the number of times that this motif was detected as modified across the entire genome.

^*c*^ No. of motifs in the genome: the number of times this motif occurs in the genome.

^*d*^ % of motifs detected: the percentage of times that this motif was detected as modified across the entire genome.

^*e*^ Mean modif. QV: the mean modification QV for all instances where this motif was detected as modified.

In our proteomic analysis, two out of the three corresponding DNA MTases, DNA MTase 1 (BAMB_HSJ1_RS01220) and DNA MTase 2 (BAMB_HSJ1_RS24645), proteins were detected at the same levels in both the parental strain and the variant at three different growth stages, while DNA MTase 3 (BAMB_HSJ1_RS09500) was not detected (Fig. S8; Table S9).

**Fig 4:**
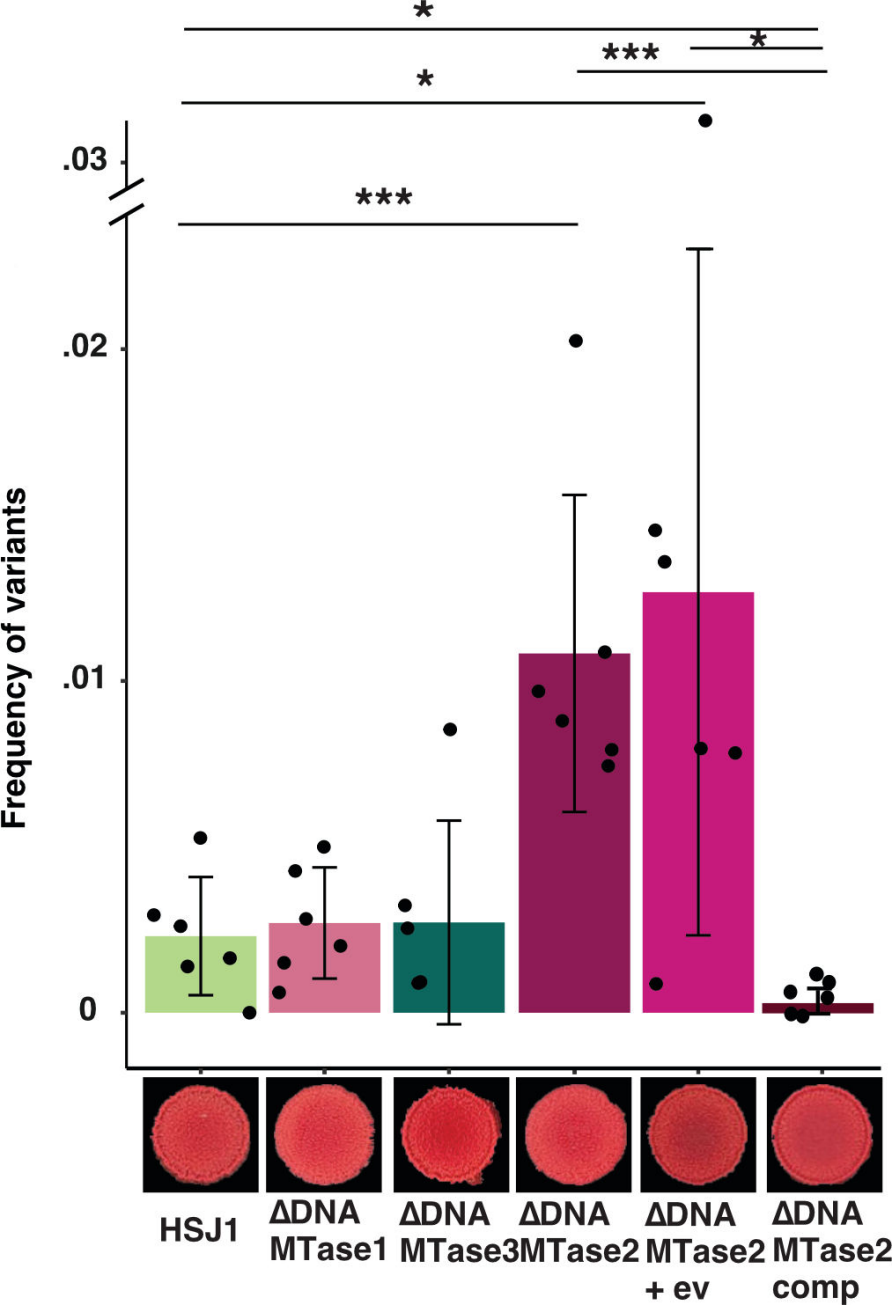
Impact of adenosine DNA MTases in *B. ambifaria* HSJ1 on phase variation. Frequency of occurrence of variant in the three DNA MTase mutants and colony morphotype on CRLA (Wilcoxon rank test). *P*-values are represented by * between 0.5 and 0.01 and *** 0.001 and 0.0001. Mean ± SD are shown in each figure. “+ ev,” empty vector and “comp,” deletion was complemented with a constitutive promotor.

To further investigate any role in the emergence of phase variants, we deleted the genes encoding all three DNA MTases in strain HSJ1 (Fig. 4; Tables S17 and S18). Importantly, supporting the hypothesis that an epigenetic factor regulates the emergence of variants, we observed a significant increase in phase variation frequency in the DNA MTase 2 mutant (responsible for the GTWW**^6^A**C motif). Moreover, overexpression of this gene in the mutant background reduced the frequency of variant emergence, even below the parental level (Fig. 4; Table S17).

Although phenotypic assays indicated no difference in biofilm formation between HSJ1 and the *∆DNAMTase 2* mutant, constitutive expression of DNA MTase 2 significantly enhanced biofilm formation (Fig. S9A). The deletion of the DNA MTase 2 gene did not affect the protease activity (Fig. S9B) but resulted in reduced swimming motility compared to the parental, likely directly due to a higher proportion of variants (Fig. S9C).

Together, these results suggest that DNA MTase 2 influences the emergence of phase variants in *Ba* and modulates phenotypes due to the higher proportion of variants.

### QS and DNA methylation do not interplay

Given the identified opposing effects of QS and DNA MTase 2 on the emergence of HSJ1v, we explored whether one could influence the other. To investigate this, we first quantified the abundance of the DNA MTase 2 protein in the HSJ1 *cepR*^-^ QS mutant compared to HSJ1 at both OD_600_ ~3 and ~5 (mid- and late-log phases, respectively, at which QS effects are easily detectable). No significant differences in abundance were detected (Fig. S10A; Table S19), which is consistent with the absence of a *cep* QS regulatory box (53) in the promoter region of the DNA MTase 2 gene.

**Fig 5:**
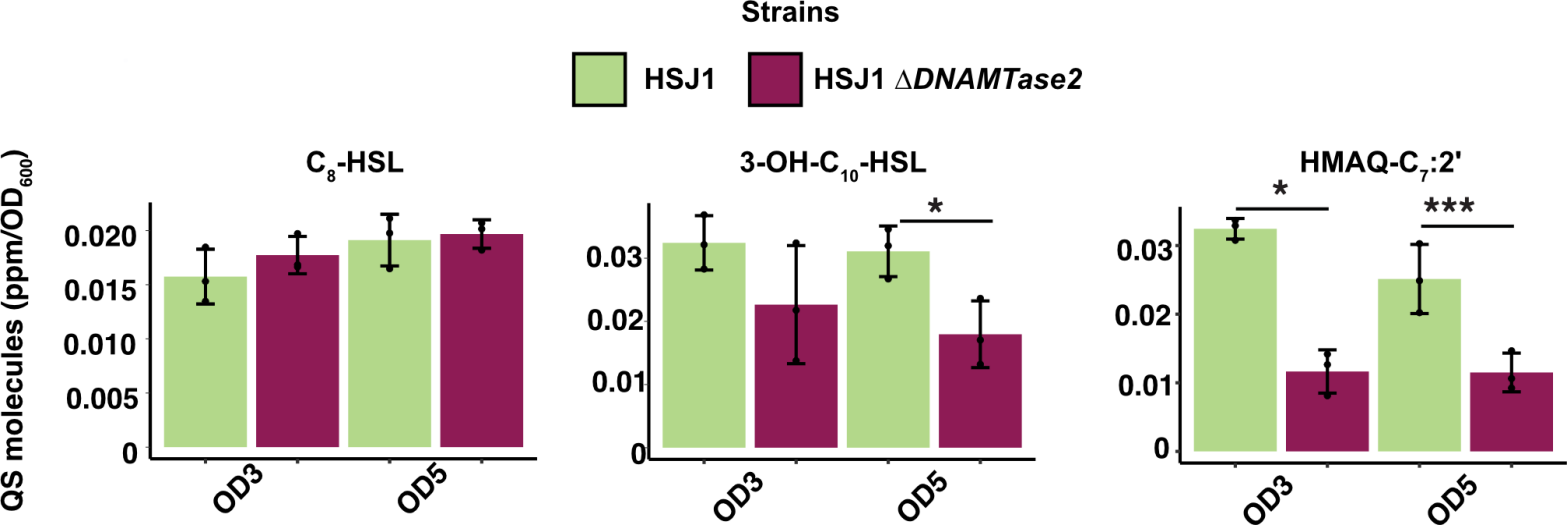
DNA MTase 2 impairs signal molecules produced by Cep2 and Hmq systems at an OD_600_ of 3 and 5. Production of AHLs and HMAQ in HSJ1 ∆DNA MTase 2 mutant (*t*-test). *P*-values are represented by * between 0.5 and 0.01, and *** 0.001 and 0.0001. Mean ± SD are shown in each figure.

Next, we quantified the abundance of proteins belonging to the Cep, Cep2, and Hmq systems, as well as their respective signal molecules: C_8_-HSL, 3-OH-C_10_-HSL, and HMAQ-C_7_:2′ between the ∆*DNAMTase2* mutant and wild-type HSJ1 (Fig. 5; Tables S19 and S20) (52). No significant differences in the abundance of CepI, CepR, CepR2, and HmqA were observed between the HSJ1 wild type and ∆*DNAMTase2* mutant. While CepI2 abundance was lower than the detection limit at OD_600_ ~3, it had the same abundance at OD_600_ ~ 5. Interestingly, the level of 3-OH-C_10_-HSL and HMAQ-C_7_:2′ was reduced in HSJ1 ∆DNAMTase2 mutant at both OD_600_ ~ 3 and ~5 (Fig. 5; Fig. S10).

While DNA MTase 2 may indirectly influence 3-OH-C_10_-HSL production, the observed reduction in HMAQ aligns with our findings that DNA MTase 2 mutant produces more variants than the parent.

## DISCUSSION

We previously reported that clinical *Ba* isolates undergo phase variation to adopt a stable colony morphotype that is more competitive in plant root colonization (22). To understand the mechanism responsible for this phase variation, we analyzed a collection of *Ba* clinical strains and found that six out of eight derived variants had lost their pC3 replicon. This aligns with previous observations of variants among environmental-, clinical-, and laboratory-adapted isolates of other Bcc species (31, 36, 44). However, the remaining two variants retain their pC3, suggesting that only these two undergo phase variation, while the other six exhibit irreversible genomic modifications.

Similar to most bacteria, Bcc species, including *Ba*, use CMV as a mechanism to modulate virulence and adapt to the dynamic host environment by reducing the production of immunogenic factors, enabling the bacteria to persist despite host immune responses and antibiotic treatments (33, 34, 82–84). Our proteomic and phenotypic analyses revealed numerous differences between *Ba* parental and variant isolates. These findings corroborate previous studies that described the effects of CMV in *Burkholderia* on secondary metabolite production and phenotypes (22, 62–68, 85–88). While the loss of pC3 explains the impact on most of the virulence factors in the variant of *Ba* strain CEP0996, no genomic variation could be found between parental *Ba* strain HSJ1 and its variant.

Given that the production of most virulence factors and secondary metabolites is controlled by QS, we quantified the production of AHL signals. Although we observed no difference in AHL levels between *Ba* HSJ1 parental and variants isolates, we observed that a *cepR^-^* mutant, which lacks a key QS transcriptional regulator, does not produce variants. This indicates that the Cep QS system activates the emergence of phase variants—this represents the first report of QS involvement in phase variation. In Bcc, and particularly in *Bc* K56-2, ShvR plays a crucial role in modulating CMV, QS, and virulence. Moreover, a naturally lower expression of *shvR* in *Bc* strain H111 leads to a smooth colony morphotype (39, 43). However, deleting *shvR* in HSJ1 does not induce changes in colony morphotype and biofilm formation and, more importantly, does not impact the emergence of phase variants. This suggests that the ShvR homolog in *Ba* HSJ1 lacks a similar role to that in *Bc*, even though its impact on replication and persistence in macrophages still needs to be investigated.

Since changes in genome sequence or QS regulation do not fully explain the phenotypic differences observed between *Ba* HSJ1 parental strain and its variant, we investigated a possible role of DNA methylation, a well-established bacterial epigenetic factor, on the emergence of phase variants (30). While the underlying mechanism for the loss of pC3 remains unknown, previous research has shown that simultaneously knocking out all three DNA MTases in *Bc* K56-2 resulted in a higher proportion of pC3-null variants (45). In our model isolates producing variants retaining the pC3 replicon, no alterations were found in the three DNA MTases, and no differences in the abundance of DNA MTase 1 and 2 were observed between the variants and their parental isolates (DNA MTase 3 was not detected). This suggests that the function of these MTases is not compromised at the genomic, transcriptional, or translational levels. However, we found that deleting the *DNA MTase 2* gene, which encodes an enzyme responsible for adenosine methylation of GTWW^6^AC DNA motifs, increases the emergence of phase variants. This demonstrated that DNA MTase 2 inhibits the emergence of phase variants, as confirmed by the almost complete loss of variants when this MTase is overexpressed.

**Fig 6:**
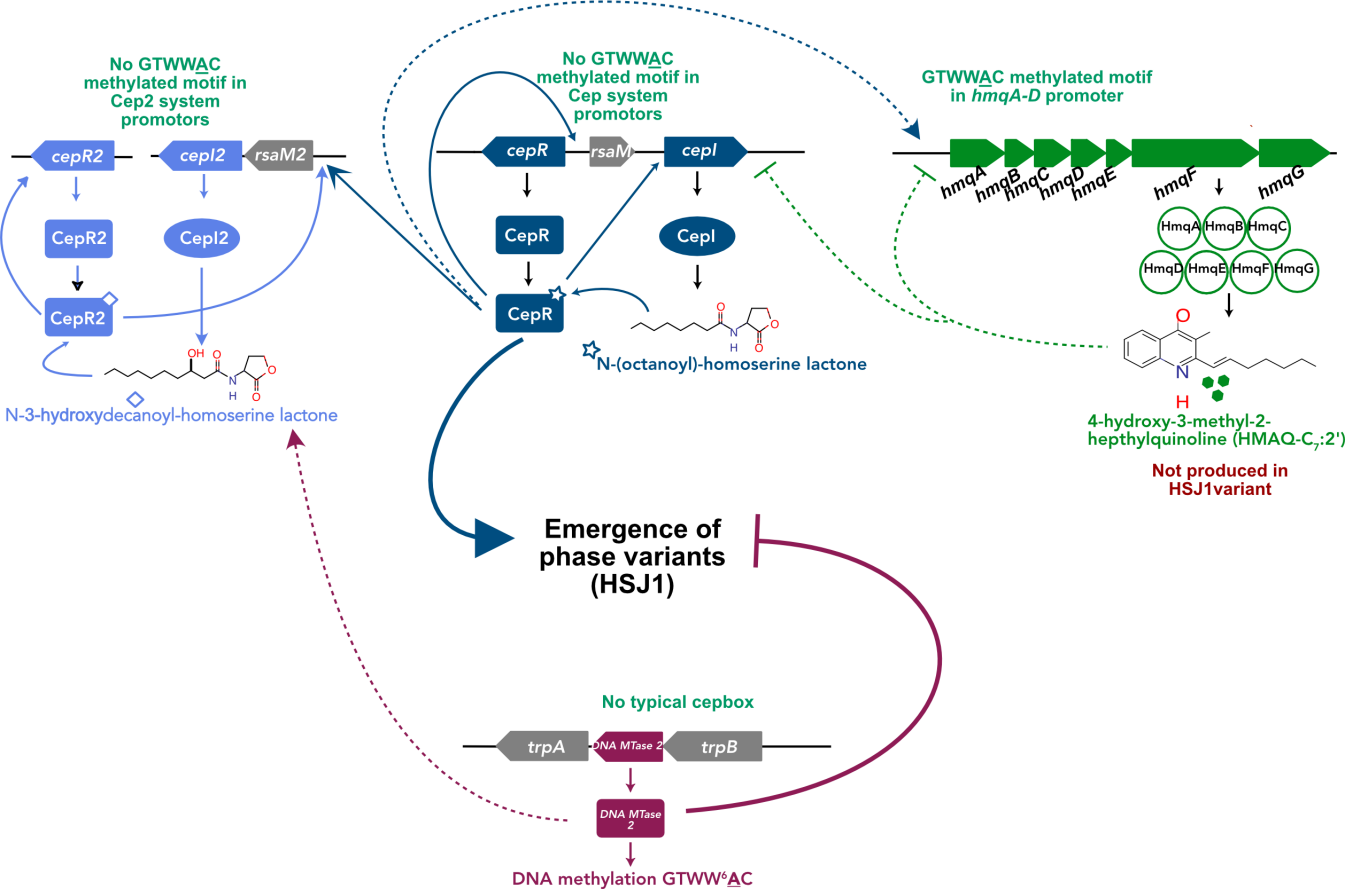
Schematic representation of the proposed regulation of phase variation in *Ba* HSJ1 Cep QS system activates phase variation in *Ba* HSJ1, while DNA MTase 2 inhibits the emergence of variants. Cep QS system does not regulate the production of DNA MTase 2 proteins. While the DNA MTase 2 does not significantly impact the abundance of proteins involved in Cep, Cep2, and Hmq systems, the production of both 3-OH-C_10_-HSL and HMAQs was reduced due to the production of variants. Confirmed regulation is indicated by a solid arrow, and proposed regulation is indicated by a dashed arrow.

Taken together, our data indicate that the Cep QS system is essential for the emergence of variants, while DNA methylation—especially DNA MTase 2—negatively impacts phase variation (Fig. 6); they probably influence gene expression by promoting or inhibiting RNA polymerase binding (46, 89). Whether these systems directly control gene transcription or act through a master phase variation regulator remains to be determined; this question will be addressed in future research.

Our results reveal an unprecedented interplay between QS and phase variation, emphasizing the complexity of bacterial rapid adaptation mechanisms and challenges in controlling the pathogenicity of *Burkholderia*. These findings also raise the possibility that similar mechanisms could exist in other bacteria. Understanding how this mechanism is triggered during infection may provide insights into *Burkholderia* adaptability, as most *Burkholderia* infections are acquired from environmental sources, with only occasional instances of patient-to-patient transmission (90–93). While a limited number of studies have reported CMV within the *Burkholderia* genus and even fewer have identified mechanisms of CMV, the impact of CMV on virulence remains significant. Future research should assess the environmental and clinical prevalence of CMV across *Burkholderia* species and uncover underlying CMV mechanisms. This could ultimately enhance our ability to combat infections caused by these highly adaptable pathogens.

## MATERIALS AND METHODS

### Screening for the presence of the third replicon in parental and variants

All strains and plasmids are listed in Tables S1 and S2, respectively. Overnight cultures were set up from −80°C glycerol stocks in Tryptic Soy broth (Bacto BD Difco, USA) at 30°C with shaking at 200 rpm. DNA was extracted from cultures following a previously described method using a Fast-Prep Beadbeater (94). PCR amplification was performed using the Easy-Taq DNA polymerase (Transgene, China) with specific primers targeting the *hisA* gene, which is present on the first chromosome, or seven different regions distributed along pC3 in *Ba*. All primers are listed in Table S3.

### Whole-genome sequencing and representation

*Ba* strains CEP0996 and HSJ1 and their respective phase variants were cultured overnight from −80°C-stored glycerol stocks in TSB at 30°C with shaking at 200 rpm. Genomic DNA was extracted from cultures using a Genomic DNA extraction kit (Transgen, China). *Ba* strains CEP0996 and HSJ1 and their respective variant’s genomes were sequenced using either Oxford Nanopore Technology (ONT, by MIGS USA) or Pacific Biosciences technology (PacBio, by Genome Quebec, Canada) RS II complemented by Illumina technology (MIGS).

HSJ1 parental and variant genomes were assembled using Unicycler (conda version 0.4) hybrid method (95) from both long and short reads. For CEP0996 parental, Unicycler hybrid (docker version 0.5), Canu version 0.0 (96), and Flye version 2.9.3-b1797 (97) assemblies were used to generate a consensus genome assembly using Trycycler version 0.5.4 (98). Then, Medaka version 1.0.3 from ONT and Polypolish version 0.5.0 (99) tools were used to polish the resulting Trycycler assembly. Each genome assembly was then annotated using BAKTA version 1.9.1 (100). Clustering of orthologous groups for annotated proteins were determined using Eggnote tool version 5 (101). To confirm the absence of the pC3 replicon in both types of variants, short reads from *Ba* CEP0996 pC3-null and *Ba* HSJ1v were mapped using minimap2 (102, 103) on their corresponding parental genomes. Coverage of reads for each chromosome was represented using one in every 100 nucleotides using Shinycircos R package (shiny::runGitHub [“shinyCircos,” “venyao”] [104]).

To assign homologous genes between CEP0996 and HSJ1, a megablastn was set up with only one output match for each gene in query using the following parameters: -evalue 10 -outfmt 6 -num_threads 3 -max_target_seqs 1 -max_hsps 1 (105).

### Comparison between genomes of *Ba* HSJ1 parental and variant

Illumina reads from *B. ambifaria* HSJ1 variant (HSJ1v) were mapped on the *B. ambifaria* HSJ1 parental genome assembly, and *B. ambifaria* HSJ1 parental Illumina reads were mapped on *B. ambifaria* HSJ1 variant genome assembly using minimap2.1 (102, 103). Then, the sorted mapped reads were used by Freebayes version 1.3.2-dirty (106) to detect any genomic variations between HSJ1 parental and HSJ1v using diploid feature and were filtered by sequencing strand bias (SPR and SAP), placement bias (EPP), variant quality (QUAL), and depth of coverage (DP) above 20 corresponding to a *P*-value below 0.01.

Both HSJ1 and HJS1v assembly were screened for inverted repeat sequences using Inverted Repeats finder tool with default settings (https://tandem.bu.edu/irf/home [107]) and for duplication regions using LASTZ software (https://github.com/lastz/lastz [108]).

### Comparative proteomic between both *Ba* CEP099 parental/variant and *Ba* HSJ1 parental/variant couples

Colonies were isolated on tryptic soy agar plates containing 0.01% Congo Red (CRTSA) following incubation at 30°C for 48 h. Cultures for both parental and variants of CEP0996 and HSJ1 were prepared in TSB and incubated overnight at 30°C with agitation in a TC Roller-drum (New Brunswick, USA) at 200 rpm. Fresh cultures were set up at an OD_600_ of 0.2 in 5 mL of TSB until the OD_600_ reached 1. Total protein was extracted as previously described (109). Briefly, after washing cell pellets with phosphate-buffered saline (PBS), frozen cells were lysed, and total proteins were precipitated with 80% ice-cold acetone.

Twenty micrograms of each sample was reduced and alkylated by 0.2 mM DTT and 0.8 mM iodoacetamide. Then, samples were digested by 0.4 µg trypsin and purified on C18 StageTips, and 1 µg was analyzed following a 120 min liquid chromatography run coupled with mass spectrometry (LC-MS/MS) on an Orbitrap Fusion Tribrid system (Thermo Fisher, USA) using data-dependant acquisition (DDA) label-free technique at the Proteomic Platform of the CHU Québec Research Center (Université de Laval, Quebec City, Canada). Raw data were searched against either CEP0996 or HSJ1 proteomes, obtained from this study against annotated genomes (Tables S4 and S5), with Fragpipe version 20 (110) using the LFQ-MBR script for label-free quantification of proteins and normalization. The resulting “combined proteins” files were filtered to remove contaminants and keep proteins for which there were at least two values for at least one group based on the label-free quantification (LFQ) intensities as each data search contains three biological replicates for each condition. Data were normalized using the variance stabilization normalization (111) and then selected to differentiate proteins with at least two missing values in one condition representing “detected/not detected.” Data containing one missing value in at least one group had the missing value imputed based on the average of the values of other replicates within the same group, and the differential protein production between each group was analyzed using a Welch’s *t*-test with Benjamini-Hochberg adjusted *P* values below 0.05 (112, 113). Analyses were performed on three biological replicates.

### Phenotypic assays

Colony morphologies were observed by spotting 15 µL from a TSB overnight culture on CRTSA plates. The plates were incubated for 2 days at 30°C.

Biofilm formation was measured by growing static cultures in 5 × 75 mm polystyrene tubes or 96-well plates. Cultures were inoculated from an overnight culture to an initial OD_600_ = 0.2 and were incubated at 30°C for 24 h without agitation. After removing unattached cells by washing, the biofilms were stained with 0.1% crystal violet, and OD_590_ was measured using a microplate plate reader (Cytation 3 or Clariostar) (22). Four technical replicates were set up for the 96-well plate experiments, and four biological replicates were used for biofilm formation in tubes. The experiment was performed three times.

Siderophore production was determined by the Chrome-Azurol S (CAS) assay (114). A five microliter bacterial suspension at OD_600_ = 5 was spotted on CAS agar plates. The plates were incubated overnight at 30°C. Areas of the siderophore production halos surrounding the colonies were then measured and reported in square centimeters. Five biological replicates were used, and the experiment was performed three times.

Protease activity was determined by spotting 15 µL of a bacterial culture at OD_600_ = 5 on 1.5% skim milk TSA plates, as previously described (53). The plates were incubated for 48 h at 30°C. Protease activity halos were then measured and reported in square centimeters. Four biological and three technical replicates were used, and the experiment was performed three times.

Bacterial flagella were observed by transmission electron microscopy. CEP0996 strains were cultured for 48 h on CRTSA plates, and HSJ1 strains were cultured in 5 mL TSB at 30°C overnight with agitation. A grid was inoculated into the liquid culture for 30 seconds and dried for 1 min. Then, cells were fixed for 1 second using a 1% paraformaldehyde solution before imaging using a Hitachi H-7100 electron microscope at the INRS-Centre Armand-Frappier Santé Biotechnologie electron microscopy platform with AMT Image Capture Engine (version 600.147).

### Determination of phase variation frequency

From single colonies, starter cultures were set up in TSB and incubated at 30°C overnight under 200 rpm shaking. The next morning, 15 µL of each culture was spotted on an LB agar plate containing 0.01% Congo Red (CRLA) and incubated for 48 h at 30°C followed by 96 h at room temperature. Each spot colony was then suspended in 1 mL PBS and serially diluted. Finally, 50 µL of the 10^6^ dilution was spread onto a CRLA plate. Three sets of dilutions were performed and plated from each colony. After incubation at 30°C, variants were counted among the total colonies per plate to determine the frequency of occurrence of variant colonies per cell per generation (115). The experiment was performed on six separate colonies and repeated twice.

### Comparative proteomics of *Ba* HSJ1, HSJ1 ∆*DNA MTase 2*, and HSJ1 *cepR^-^* strains

Concerning sample preparation for proteomics to compare abundances of proteins of interest between HSJ1 parental, HSJ1 *cepR*^-^, and HSJ1 ∆DNAMTase2, samples were cultured as previously described until the OD_600_ reached ~3 and 5. Total proteins were extracted by lysing the cells in 1% SDC in 100 mM HEPES pH 8.5. Then, the abundance of proteins was quantified using a BCA assay, and 20 µg of proteins was trypsin digested and cleaned on one SBD-RPS disk to normalize proteins to 10 µg for each sample. Using an Acquity M-class nanoLC system (Waters, USA), five microliters of the sample was loaded at 15 µL/min for 3 min onto a nanoEase Symmetry C18 trapping column (180 µm × 20 mm) before being washed onto a PicoFrit column (75 µm × 350 mm; New Objective, Woburn, MA, USA) packed with SP-120-1.7-ODS-BIO resin (1.7 µm, Osaka Soda Co., Japan) heated to 45°C. Peptides were eluted from the column and into the source of a Q Exactive Plus mass spectrometer (Thermo Scientific, USA) using the following program: 5%–30% MS buffer B (98% acetonitrile +0.2% formic acid) over 90 min, 30%–80% MS buffer B over 3 min, 80% MS buffer B for 2 min, and 80%–5% for 3 min. The eluting peptides were ionized at 2,400 V. A DDA MS/MS experiment was performed, with a survey scan of 350–1,500 Da performed at 70,000 resolution for peptides of charge state 2+ or higher with an AGC target of 3e6 and a maximum injection time of 50 ms. The top 12 peptides were selected and fragmented in the HCD cell using an isolation window of 1.4 *m/z*, an AGC target of 1e5, and a maximum injection time of 100 ms. Fragments were scanned in the Orbitrap analyzer at 17,500 resolution, and the product ion fragment masses were measured over a mass range of 120–2,000 Da. The mass of the precursor peptide was then excluded for 30 seconds. Raw data search was done against HSJ1 proteome without a match between runs. Data analysis was proceeded as described above using LFQ intensities.

### Quantification of signal molecules

AHLs and HMAQs were extracted from 4 mL cultures with ethyl acetate (1:1) and concentrated 10 times in acetonitrile, as previously described (52, 53, 116). 5,6,7,8-tetradeutero-4-hydroxy-2-heptylquinoline was used as an internal standard. Samples were analyzed by LC-MS/MS in positive electrospray ionization using a Kinetex 5-mm EVO C18 100-Å 100- by 3-mm reverse-phase column. A Quattro Premier XE triple quadrupole was used as the detector (Waters, USA). A multiple reaction monitoring program was used to detect HMAQ families. This experiment was conducted with three independent biological replicates.

### DNA methylome of *Ba* HSJ1 parental

PacBio sequencing data were further analyzed for DNA methylation motifs in both HSJ1 and HSJ1v only, as it was determined on extracted DNA from one single overnight culture. All “bax.h5” PacBio RSII files were converted to one movie bam file using bax2bam tool (https://github.com/PacificBiosciences). Then, two movie.subreads.bam files were merged to generate one SMRT cell for each DNA sample using pbcoretools (https://github.com/PacificBiosciences). Both created SMRT cells were mapped to *Ba* HSJ1 genome assembly using BLASR (117). Alignment files were then sorted, and an index file was created for the reference genome fasta and alignment files by samtools (118). DNA modifications were identified using kinetictools (https://github.com/PacificBiosciences) based on a mapQvThreshold of 30. Note that motifmaker-master (https://github.com/PacificBiosciences) can be used to change the mapQvThreshold to redo a faster analysis (119, 120).

### Mutant construction in *Ba* HSJ1 parental

Single mutants of BAMB_HSJ1_RS01220 (CAC^**6**^**A**G motif named as DNA MTase 1), BAMB_HSJ1_RS24645 (GTWW^**6**^**A**C motif named as DNA MTase 2), and BAMB_HSJ1_RS09500 (RG**^6^A**TCY motif named as DNA MTase 3) putative DNA MTase-encoding genes and BAMB_HSJ1_RS29045 (putative *shvR*) were generated using homologous recombination with the pEX18Tet-PheS allelic replacement plasmid (121). Sequences of 5′ UTR and 3′ UTR both including nine nucleotides of the encoding sequence of targeted genes and trimethoprim resistance gene were amplified with flanking regions by PCR using specific primers (Table S3) and EasyTaq polymerase (Transgen, China) or Phusion HiFi polymerase (Thermofisher, Australia). PCR-amplified inserts and the pEX18Tet-PheS previously digested by BamHI and PstI (NEB, Australia or Thermofisher, Canada) were assembled using the pEasy-Uni Seamless Cloning and Assembly kit (Transgen, China) or NEBuilder (NEB, Australia) method and transformed into *Escherichia coli* Trans-T1 (Transgen, China) or *E. coli* NEB5ɑ (NEB, Australia). The resulting plasmids were transformed into conjugative *E. coli* SM10 to transfer each plasmid into HSJ1 using bi-parental conjugation. Then, tetracycline-resistant recombinants were selected on TSA with 250 µg/mL tetracycline and spread on M9 agar plates containing 100 µg/mL trimethoprim and 0.1% chlorophenylalanine (Sigma, USA) to cure pEX18Tet-PheS. The resulting secondary recombinants were transformed with the flipase-producing pFLe4 plasmid to remove the trimethoprim resistance gene. Selection of colonies was obtained with 300 µg/mL kanamycin and incubated at 30°C. The resulting colonies were screened on an LB agar plate containing 250 µg/mL trimethoprim to select trimethoprim-sensitive mutants (Table S1). Loss of the trimethoprim cassette was confirmed by PCR.

### Complementation of DNA MTase genes in *Ba* HSJ1 mutants

Each putative DNA MTase-encoding gene was amplified using EasyTaq (Transgen, China) or Phusion HiFi polymerase (Thermofisher, Australia), and KpnI and HindIII restriction sites were added in the primers used. The pMLS7 plasmid encoding for a constitutive S7 promotor was digested by KpnI and HindIII enzymes (NEB, Australia). Both the resulting PCR fragment and digested plasmid were purified on an agarose gel and ligated using the T4 DNA ligase (NEB, Australia). The resulting DNA MTase complementation vectors were transformed into *E. coli* SM10 and transferred by bi-parental conjugation into the different *Ba* HSJ1 DNA MTase mutants (Table S1).

## Data Availability

Genomes from this study are available in GenBank under the BioProject: PRJNA896258 (121, 122). Proteomic raw data were deposited to the ProteomeXchange Consortium via the PRIDE partner repository with the data set identifier PXD055955 (123, 124).

## SUPPLEMENTAL MATERIAL

Supplemental figures jb.00531-24-s0001.pdf Fig. S1 to S10.

Supplemental tables jb.00531-24-s0002.xlsx Tables S1 to S20.

ASM does not own the copyrights to Supplemental Material that may be linked to, or accessed through, an article. The authors have granted ASM a non-exclusive, world-wide license to publish the Supplemental Material files. Please contact the corresponding author directly for reuse.
