# Supplementary material for: Quorum sensing and DNA methylation play active roles in clinical Burkholderia phase variation
Source: J Bacteriol. 2025 Feb 14;207(3):e00531-24. doi: 10.1128/jb.00531-24 (PMC11925244; doi:10.1128/jb.00531-24)
Supplement: Supplemental figures — Fig. S1 to S10. [file jb.00531-24-s0001.pdf]

Supplemental Material

Quorum sensing and DNA methylation play active roles in clinical *Burkholderia* phase variation

Pauline M.L. Coulon[1,2,3], Marie-Christine Groleau[2], Abderrahman Hachani[3], Matthew P. Padula[4], Timothy P. Stinear[3,5] & Eric Déziel[2]

[1]Australian Institute for Microbiology and Infection, Faculty of Science, University of Technology Sydney, NSW, Australia

[2]Centre Armand-Frappier Santé Biotechnologie, Institut National de la Recherche Scientifique (INRS), Laval, QC, Canada

[3]Department of Microbiology and Immunology, Doherty Institute, University of Melbourne, Victoria, Australia

[4]School of Life Sciences, Faculty of Science, University of Technology Sydney, NSW, Australia

[5]Centre for Pathogen Genomics, University of Melbourne, Victoria, Australia

Corresponding authors:

Pauline M.L. Coulon, Australian Institute for Microbiology and Infection, Faculty of Science, University of Technology Sydney, NSW, Australia, +61390353555, pauline.coulon@uts.edu.au

Eric Déziel, Centre Armand-Frappier Santé Biotechnologie, Institut National de la Recherche Scientifique (INRS), Laval, QC, Canada, +14506875010, eric.deziel@inrs.ca

A

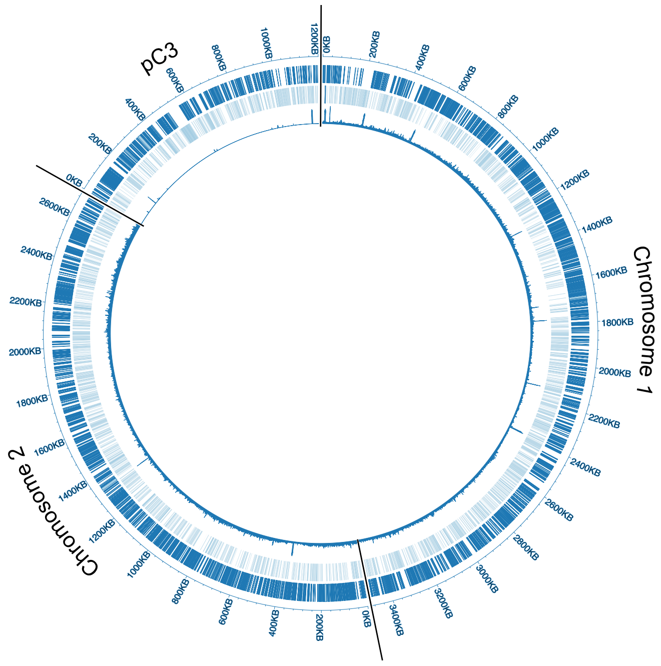

B

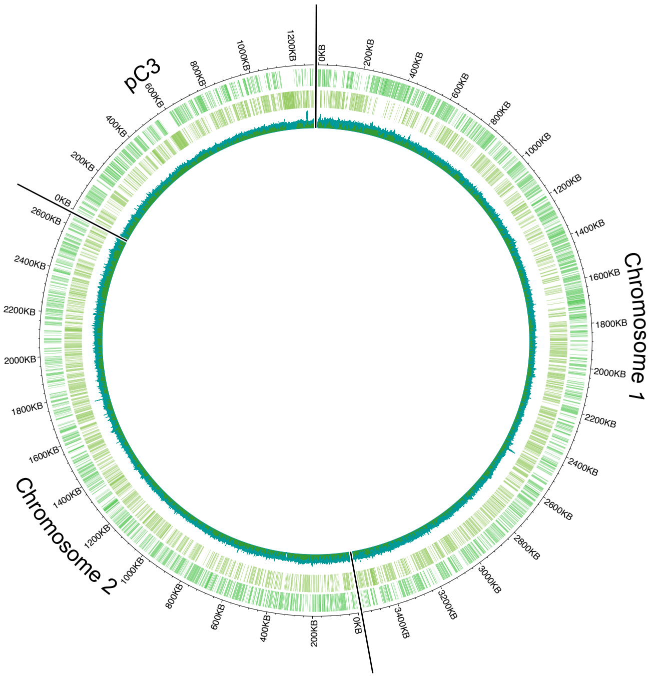

Figure S1. Genomic profiles of both types of *B. ambifaria* colony morphotype variants

A) Illumina reads from *Ba* CEP0996 pc3-null DNA were mapped to its respective *Ba* CEP0996 WT assembled genome. B) Illumina reads from *Ba* HSJ1 pc3-positive DNA were mapped to its respective *Ba* HSJ1 WT assembled genome. The first inside circle represents genes on the positive strand of DNA; the second circle brand represents genes on the negative strand of DNA; the innermost circle represents the coverage of the Illumina reads every 100 bp. Black lines were added to represent the separation of each chromosome.

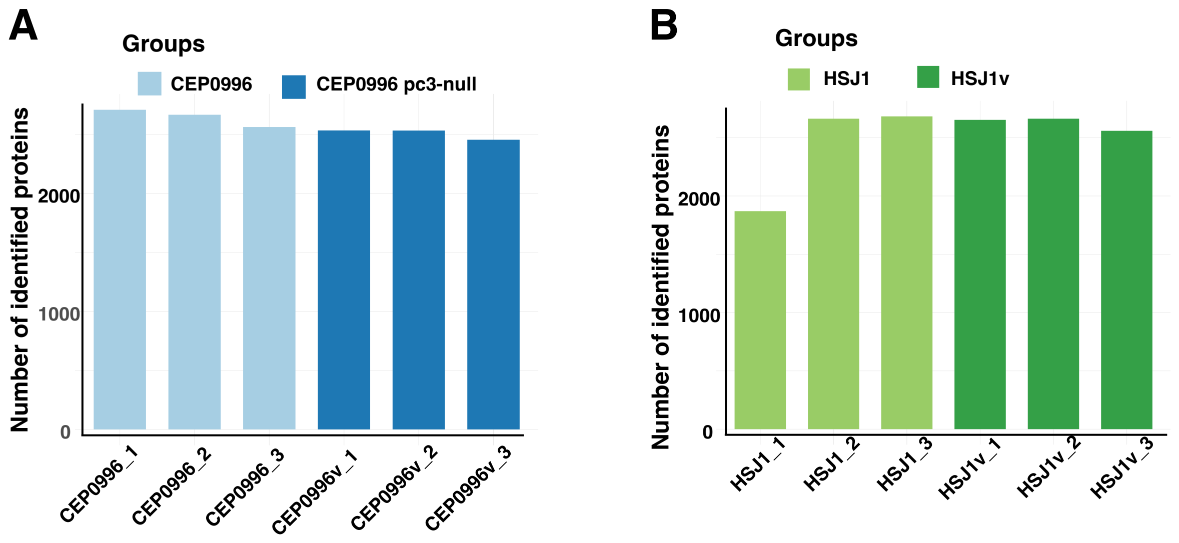

Figure S2. Number of identified proteins across CEP0996 and HSJ1 wildtype and variant proteomes.

A) Identified proteins in CEP0996 proteomes B) Identified proteins in HSJ1 proteomes. *Note: HSJ1 replicate one has a lower number of identified proteins explaining its difference between the two other replicates. However, after normalization and imputation of the selected data (refer to method section), the median of intensities is similar for all samples and the box plots display a similar repartition of the intensities across all samples.*

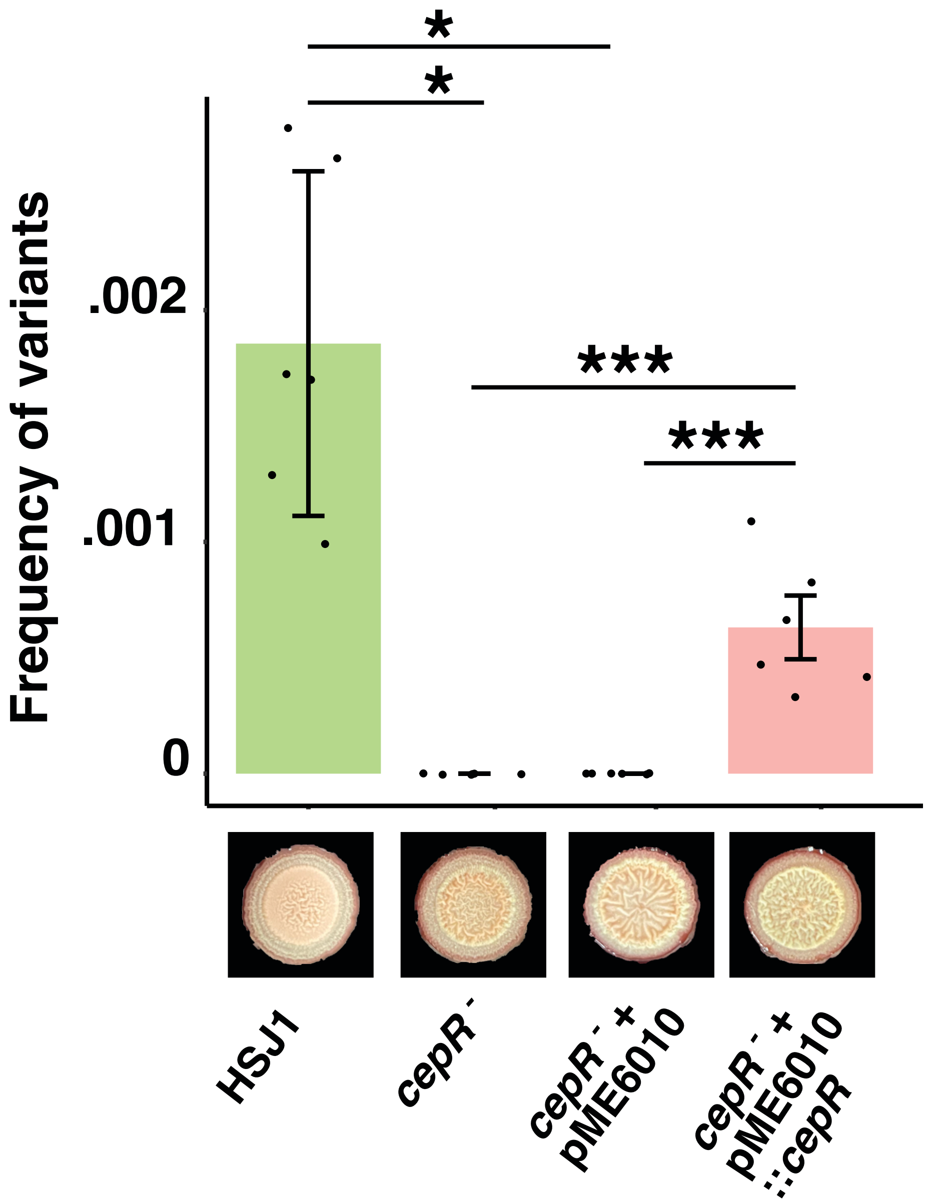

Figure S3. ***Ba*** **HSJ1 quorum sensing is involved in phase variation**

Frequency of variants were calculated in WT, *cepR*-, *cepR*⁻ + pME6000 and *cepR*⁻ + pME6000::*cepR*. WT was compared to the other strains by a t-test, as well as cepR⁻+ pME6000::*cepR* and other strains. P-values are represented by * between 0.5 and 0.01, *** 0.001 and 0.0001. Mean ± SD are shown on each figure.

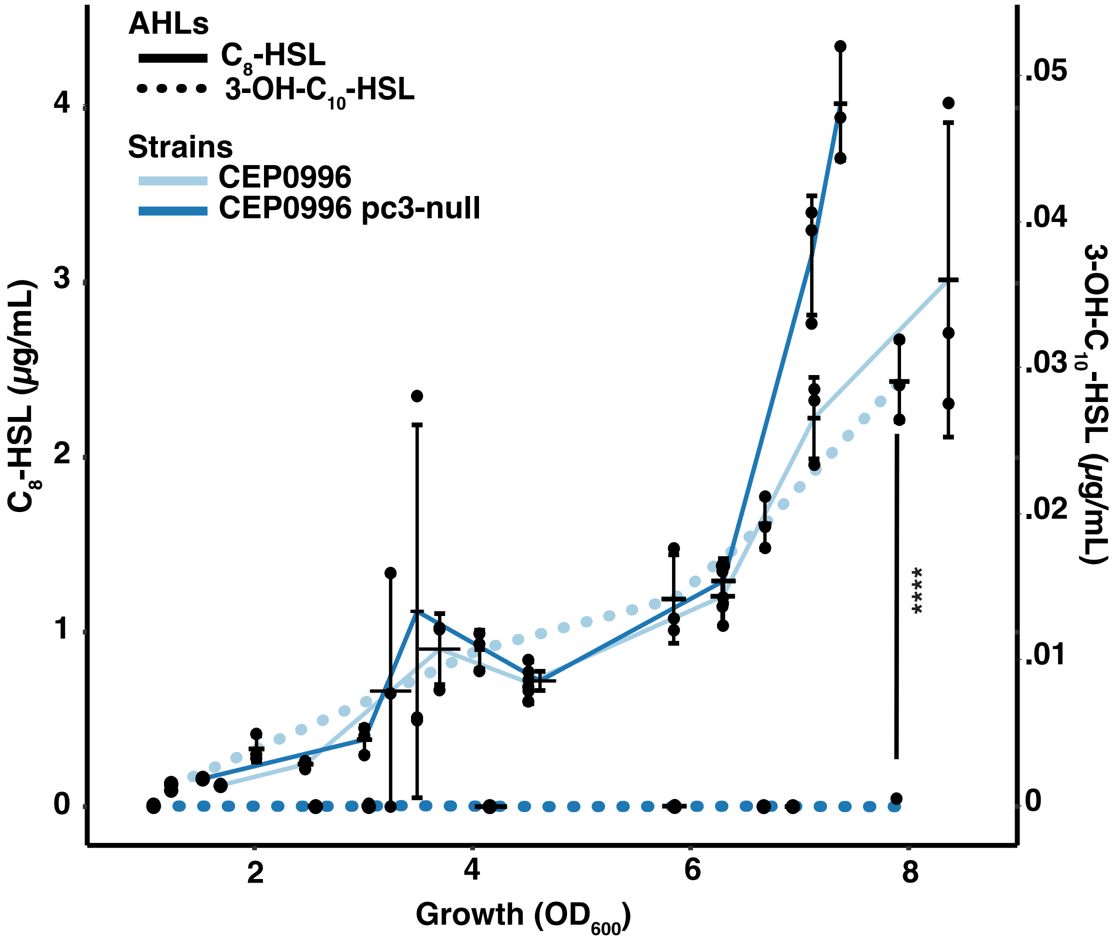

Figure S4. only Cep2 QS is impaired in *Ba* CEP0996v compared to WT

Production of AHLs in *Ba* CEP0996 WT and Variant (ANOVA). P-values are represented by *** 0.001 and 0.0001. Mean ± SD are shown on each figure.

```
Consensus   MANVRLAKRLRGMPSLDFLRGFECAARHLSFTRAGQELNVTQSAVSRQVKALEEQLRIEL   60
shvR HSJ1   ............................................................   60
shvR AMMD   ............................................................   60
shvR J2315  ...........SL...............................................   60

Consensus   FHRHIRSLTLTDKGRELYDAISIALSDLESVVGKLSSSVGQRSISLSTTVSFAALWLIPR  120
shvR HSJ1   ............................................................  120
shvR AMMD   ............................................................  120
shvR J2315  ......................F...................A.................  120

Consensus   LGSFRASYPDIDVRVSATSEIEDLKRKRLHLAVRYAGPCTPLDDTQVLFRERVVAVCSPA  180
shvR HSJ1   ............................................................  180
shvR_AMMD   ............................................................  180
shvR J2315  ......................................Y.ST...D.............S  180

Consensus   LATAVGGRPSMTPDDLDKHVLLHLDDPRGEWPWYAWSNLLKALGVPRLRPVGALHFSQYD  240
shvR HSJ1   ............................................................  240
shvR AMMD   ............................................................  240
shvR J2315  .L..M.DAA..K.A...................HG..H...E........A.........  240

Consensus   QLVQAAVDGHGIAIGRRPLIDGLLKQGRLVELFPHCTVASGSYAVVQNPDACNEFDIAVL  300
shvR HSJ1   ............................................................  300
shvR AMMD   ............................................................  300
shvR J2315  ...................V.......................VL...............  300

Consensus   TNWLLDEASVPPQAPPDNAGSNVLPLYRVG*                               331
shvR HSJ1   ...............................                               331
shvR AMMD   ...............................                               331
shvR J2315  ......Q.CPALAPEQ.T.......M.....                               331
```

Figure S5. Amino acid alignment of *ShvR*, a LysR regulator known to regulate QS and virulence in Bc

Alignment of ShvR amino acid sequences between *Ba* HSJ1, *Ba* AMMD and *Bc* J2315. The alignment was made using ClustalOmega (v 1.2.4).

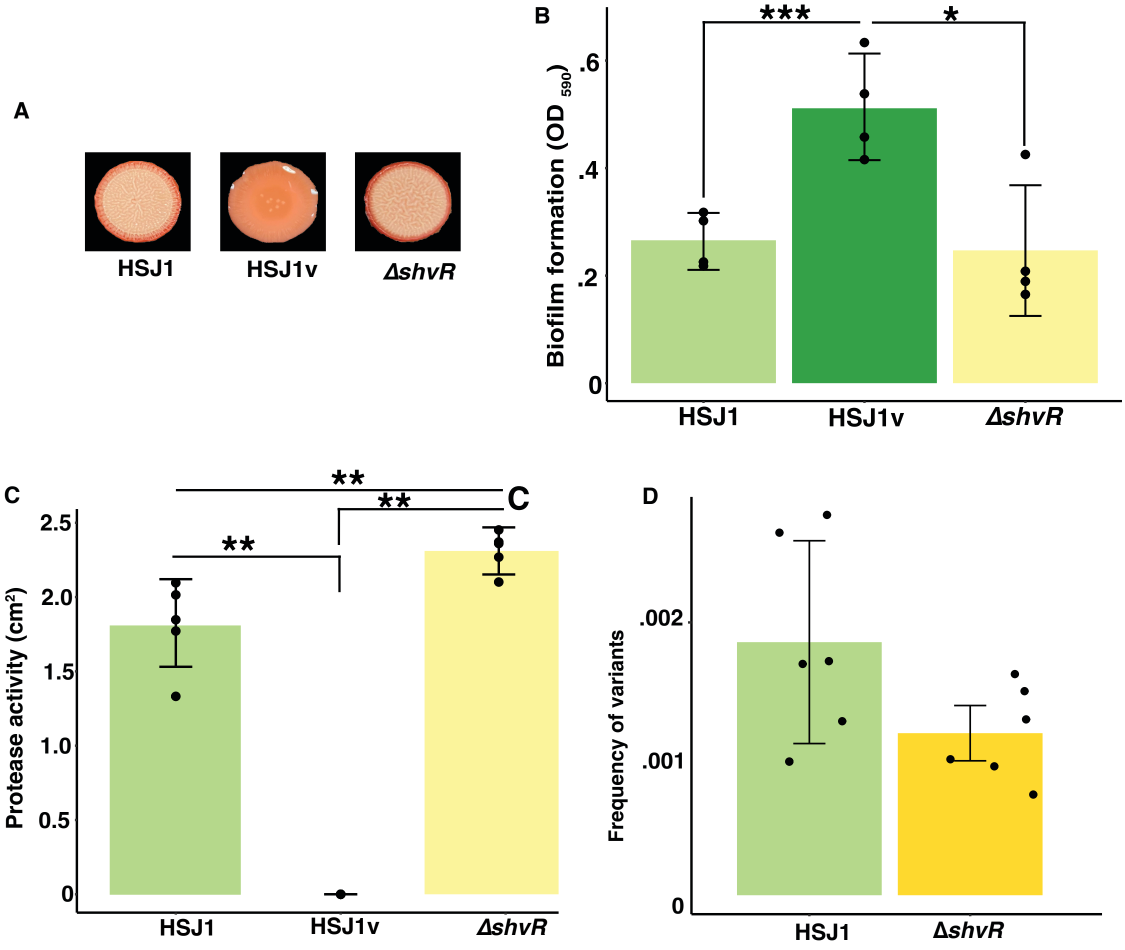

Figure S6. ShvR does not regulate the same phenotypes in *Ba* HSJ1 as in *Bc* K56-2

A) Colony morphotype on CRLA. B) Biofilm formation (t-test). C) Protease activity (Wilcoxon rank test). D) Frequency of occurrence of variants in Δ*shvR* (t-test). P-values are represented by * between 0.5 and 0.01, ** between 0.01 and 0.001, *** 0.001 and 0.0001. Mean ± SD are shown on each

DNA MTase 1

```
Consensus                        MQKLDAESPQAMSTDFIADNVARLKALFPELVTEGPDGASVDVDVLKALVGERTVGAADE   60
CEP0996_DNA_MTase1 translation   ............................................................   60
CEP0996v_DNA_MTase1 translation ............................................................   60
HSJ1_DNA_MTase1 translation      ............................................................   60
HSJ1v_DNA_Mtase1 translation     ............................................................   60

Consensus                        RYGFHWHGKRSARQAALTPSTGTLRPCPDDSVAWDDTRHLVIEGDNLDVMKLLHKSYAGK  120
CEP0996_DNA_MTase1 translation   ............................................................  120
CEP0996v_DNA_MTase1 translation ............................................................  120
HSJ1_DNA_MTase1 translation      ............................................................  120
HSJ1v_DNA_Mtase1 translation     ............................................................  120

Consensus                        VKLVYIDPPYNTGSDFVYPDDFSDSIRHYLAMTGQTDGGVKRSTNTEANGRFHTDWLNMM  180
CEP0996_DNA_MTase1 translation   ............................................................  180
CEP0996v_DNA_MTase1 translation ............................................................  180
HSJ1_DNA_MTase1 translation      ............................................................  180
HSJ1v_DNA_Mtase1 translation     ............................................................  180

Consensus                        YPRLKLAHALLSDEGLIAVHIDEHEVHALVLMLREIFGEENELGVAVWDKRNPKGDARGV  240
CEP0996_DNA_MTase1 translation   ............................................................  240
CEP0996v_DNA_MTase1 translation ............................................................  240
HSJ1_DNA_MTase1 translation      ............................................................  240
HSJ1v_DNA_Mtase1 translation     ............................................................  240

Consensus                        AYQHESLVLFARNAETLLEQAPLKRPKRNAQRMLDAAHDAVYRSGNAKDAQKAYRAWMKA  300
CEP0996_DNA_MTase1 translation   ............................................................  300
CEP0996v_DNA_MTase1 translation ............................................................  300
HSJ1_DNA_MTase1 translation      ............................................................  300
HSJ1v_DNA_Mtase1 translation     ............................................................  300

Consensus                        QTNLSGGEVMYDRLSADGRVYRLVSMAWPNKKKAPEEYFTPLIHPVTGKPCAMPARGWRN  360
CEP0996_DNA_MTase1 translation   ............................................................  360
CEP0996v_DNA_MTase1 translation ............................................................  360
HSJ1_DNA_MTase1 translation      ............................................................  360
HSJ1v_DNA_Mtase1 translation     ............................................................  360

Consensus                        PPATMQALIERGQIEFGPDETTQPQRIYYLDENMYENVPSVLPFAGSDDALLKTLGIPFD  420
CEP0996_DNA_MTase1 translation   ............................................................  420
CEP0996v_DNA_MTase1 translation ............................................................  420
HSJ1_DNA_MTase1 translation      ............................................................  420
HSJ1v_DNA_Mtase1 translation     ............................................................  420

Consensus                        QPKPVDFAAAVIGWCTRGDDIVLDCFAGSGSTGHAVMQVNATDGGARRYILVQLPETLDR  480
CEP0996_DNA_MTase1 translation   ............................................................  480
CEP0996v_DNA_MTase1 translation ............................................................  480
HSJ1_DNA_MTase1 translation      ............................................................  480
HSJ1v_DNA_Mtase1 translation     ............................................................  480

Consensus                        RDKTQLAAADFCAKLKKPTTLAEITKERLRRAAQQVARDYPESYGDLGFRVYRLDTTNVI  540
CEP0996_DNA_MTase1 translation   ............................................................  540
CEP0996v_DNA_MTase1 translation ............................................................  540
HSJ1_DNA_MTase1 translation      ............................................................  540
HSJ1v_DNA_Mtase1 translation     ............................................................  540

Consensus                        EWDPRRDDFDHALFASVEHVKTGRSEDDLLAELTLKLGLDLCTPVEHHXVAGKTVHLIGR  600
CEP0996_DNA_MTase1 translation   ................................................R...........  600
CEP0996v_DNA_MTase1 translation ................................................R...........  600
HSJ1_DNA_MTase1 translation      ................................................W...........  600
HSJ1v_DNA_Mtase1 translation     ................................................W...........  600

Consensus                        SIVACFDARISRDDAGPLSDGIVELLDATGATRDVTCLFRDSGFVDDVAKLNLAALLEQH  660
CEP0996_DNA_MTase1 translation   ............................................................  660
CEP0996v_DNA_MTase1 translation ............................................................  660
HSJ1_DNA_MTase1 translation      ............................................................  660
HSJ1v_DNA_Mtase1 translation     ............................................................  660

Consensus                        GVKRVRSL*                                                     669
CEP0996_DNA_MTase1 translation   .........                                                     669
CEP0996v_DNA_MTase1 translation .........                                                     669
HSJ1_DNA_MTase1 translation      .........                                                     669
HSJ1v_DNA_Mtase1 translation     .........                                                     669
```

DNA MTase 2

```
Consensus                                   MRDLIEEPGGGAASEAEAGQPAVAVPCALPSGIELHNRDFMTEAARLPDASIDLIVADPP   60
CEP0996_DNA_MTase2 translation              ............................................................   60
CEP0996v_DNA_Mtase2 (reversed) translation ............................................................   60
HSJ1_DNA_MTase2 translation                 ............................................................   60
HSJ1v_DNA_Mtase2 translation                ............................................................   60

Consensus                                   YGLGKDYGNDSDKRSGDDFLAWTREWLELAIPKLKPSGSMYIFCTWQYAPEIFSFLKTQL  120
CEP0996_DNA_MTase2 translation              ............................................................  120
CEP0996v_DNA_Mtase2 (reversed) translation ............................................................  120
HSJ1_DNA_MTase2 translation                 ............................................................  120
HSJ1v_DNA_Mtase2 translation                ............................................................  120

Consensus                                   TMVNEIIWDRRVPSMGGTTRRFTSVHDNIGFFAVSKAYYFDLDPVRIPYDADTKKARSRK  180
CEP0996_DNA_MTase2 translation              ............................................................  180
CEP0996v_DNA_Mtase2 (reversed) translation ............................................................  180
```

```
HSJ1_DNA_MTase2 translation                ............................................................  180
HSJ1v_DNA_Mtase2 translation               ............................................................  180

Consensus                                  LFEGSKWLEMGYNPKDVWSVSRLHRQHAERVDHPTQKPLEIIERMVLASCPPGGRVLDPF  240
CEP0996_DNA_MTase2 translation             ............................................................  240
CEP0996v_DNA_Mtase2 (reversed) translation ............................................................  240
HSJ1_DNA_MTase2 translation                ............................................................  240
HSJ1v_DNA_Mtase2 translation               ............................................................  240

Consensus                                  MGSGTTAVACARQGRDFVGYEINESYCAIAHERVNALAAQACA*                  284
CEP0996_DNA_MTase2 translation             ............................................                  284
CEP0996v_DNA_Mtase2 (reversed) translation ............................................                  284
HSJ1_DNA_MTase2 translation                ............................................                  284
HSJ1v_DNA_Mtase2 translation               ............................................                  284
```

DNA MTase 3

```
Consensus                                  MANPIIPWIGGKRRLADHJIPRFPXHDCYVEVFAGGAALYFLRPPAKVEVINDVNGELIN   60
CEP0996_DNA_MTase3 translation             ..................L.....A...................................   60
CEP0996v_DNA_Mtase3 (reversed) translation ..................L.....A...................................   60
HSJ1_DNA_MTase3 translation                ..................I.....S...................................   60
HSJ1v_DNA_Mtase3 translation               ..................I.....S...................................   60

Consensus                                  LYRVVQHHXEEFVRQFKWALTSRQVFEWLKQTXPETLTDIQRAARFYYLQKSCFGAKLEG  120
CEP0996_DNA_MTase3 translation             ........M.......................A...........................  120
CEP0996v_DNA_Mtase3 (reversed) translation ........M.......................A...........................  120
HSJ1_DNA_MTase3 translation                ........L.......................V...........................  120
HSJ1v_DNA_Mtase3 translation               ........L.......................V...........................  120

Consensus                                  QSFGTATTTPPGLNLLRIEEXLSAAHLRLANTFIERLDWAACIDRYDRXHTLFYLDPPYY  180
CEP0996_DNA_MTase3 translation             ....................D...........................T...........  180
CEP0996v_DNA_Mtase3 (reversed) translation ....................D...........................T...........  180
HSJ1_DNA_MTase3 translation                ....................E...........................P...........  180
HSJ1v_DNA_Mtase3 translation               ....................E...........................P...........  180

Consensus                                  ETEGYGVAFPFXEYEKMAXRLRSJKGRAIVSLNDHPDIRRXFBGFXIETVPIQYTVGGGX  240
CEP0996_DNA_MTase3 translation             ...........S......Q....I................V.D..H.............G  240
CEP0996v_DNA_Mtase3 (reversed) translation ...........S......Q....I................V.D..H.............G  240
HSJ1_DNA_MTase3 translation                ...........G......H....L................A.N..Y.............-  239
HSJ1v_DNA_Mtase3 translation               ...........G......H....L................A.N..Y.............-  239

Consensus                                  XXXERNELIIXSWDDAAQPVGLF*                                      264
CEP0996_DNA_MTase3 translation             RAA.......F.............                                      264
CEP0996v_DNA_Mtase3 (reversed) translation RAA.......F.............                                      264
HSJ1_DNA_MTase3 translation                KGV.......Y.............                                      263
HSJ1v_DNA_Mtase3 translation               KGV.......Y.............                                      263
```

Figure S7. Amino acid alignments of the three DNA MTases from both CEP0996 and HSJ1 parental and variant strains using Clustal Omega.

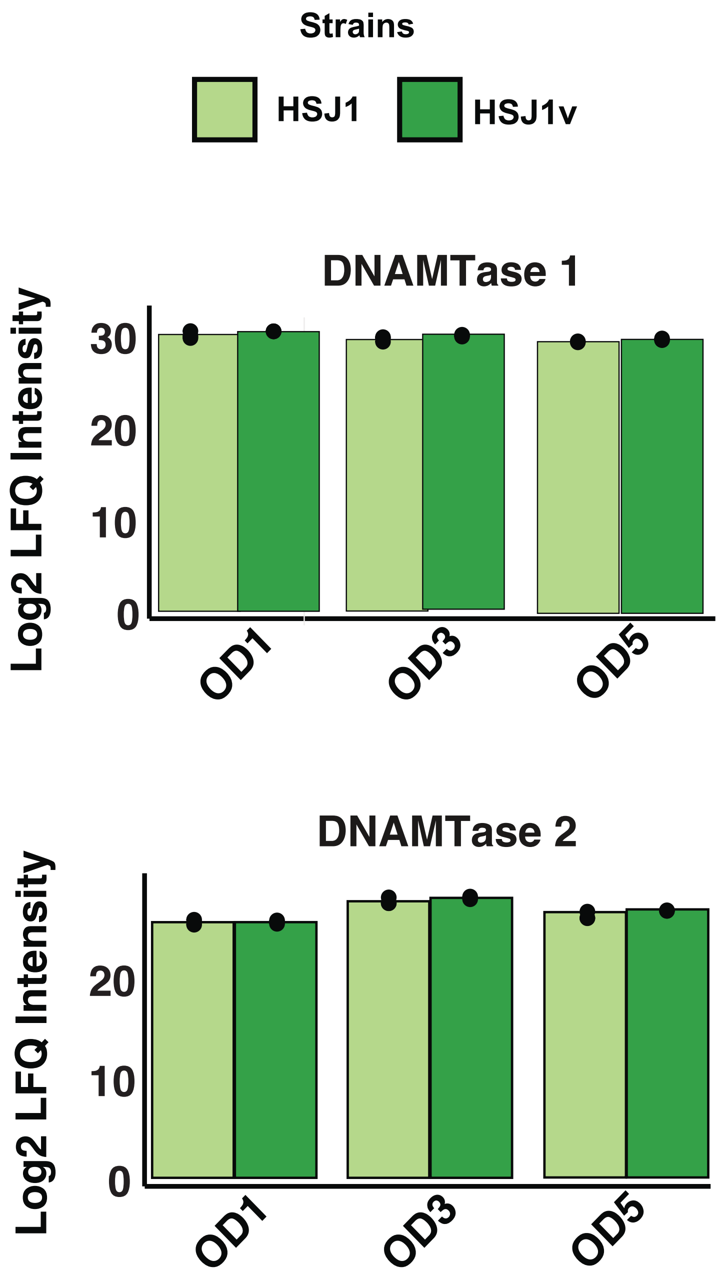

Figure S8. Protein abundance of DNA MTases 1 and 2 are equivalent in HSJ1v compared to HSJ1

Quantified abundance of protein of DNA MTases 1 and 2 in HSJ1 and HSJ1v. Log_2 fold change is represented on the graph. A false discovery rate (FDR) of 0.01 adjusted p value t-test has been performed. Mean and individual replicate are shown on each figure. Three biological replicates were run.

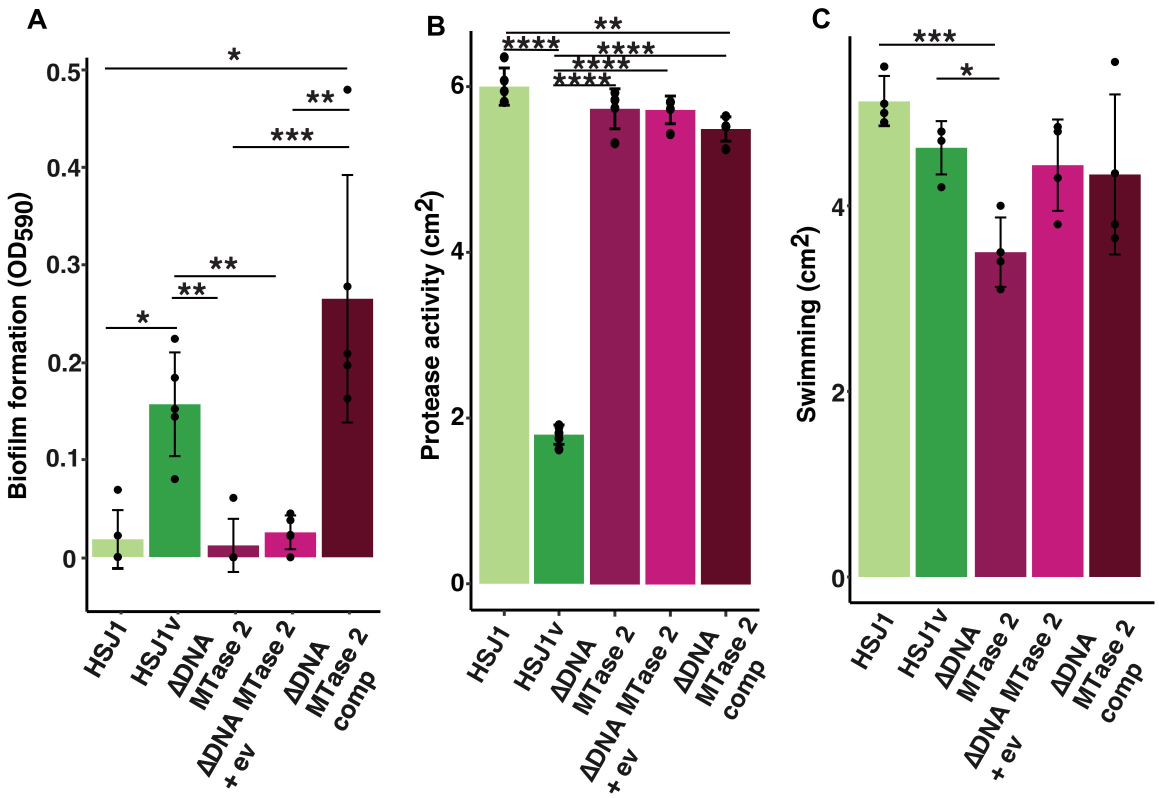

Figure S9. DNA MTase 2 does not impact biofilm formation, swimming motility and proteolytic activity.

A) Biofilm formation (Kruskal Wallis test with Dunn test posthoc). B) Protease production (Anova with Tukey multiple comparisons). C) Swimming motility (ANOVA test with Tukey posthoc). P-values are represented by * between 0.5 and 0.01, ** between 0.01 and 0.001, *** 0.001 and 0.0001, and **** inferior to 0.0001. Mean ± SD are shown on each figure. "+ ev" means empty vector, "comp" means deletion was complemented with a constitutive promotor.

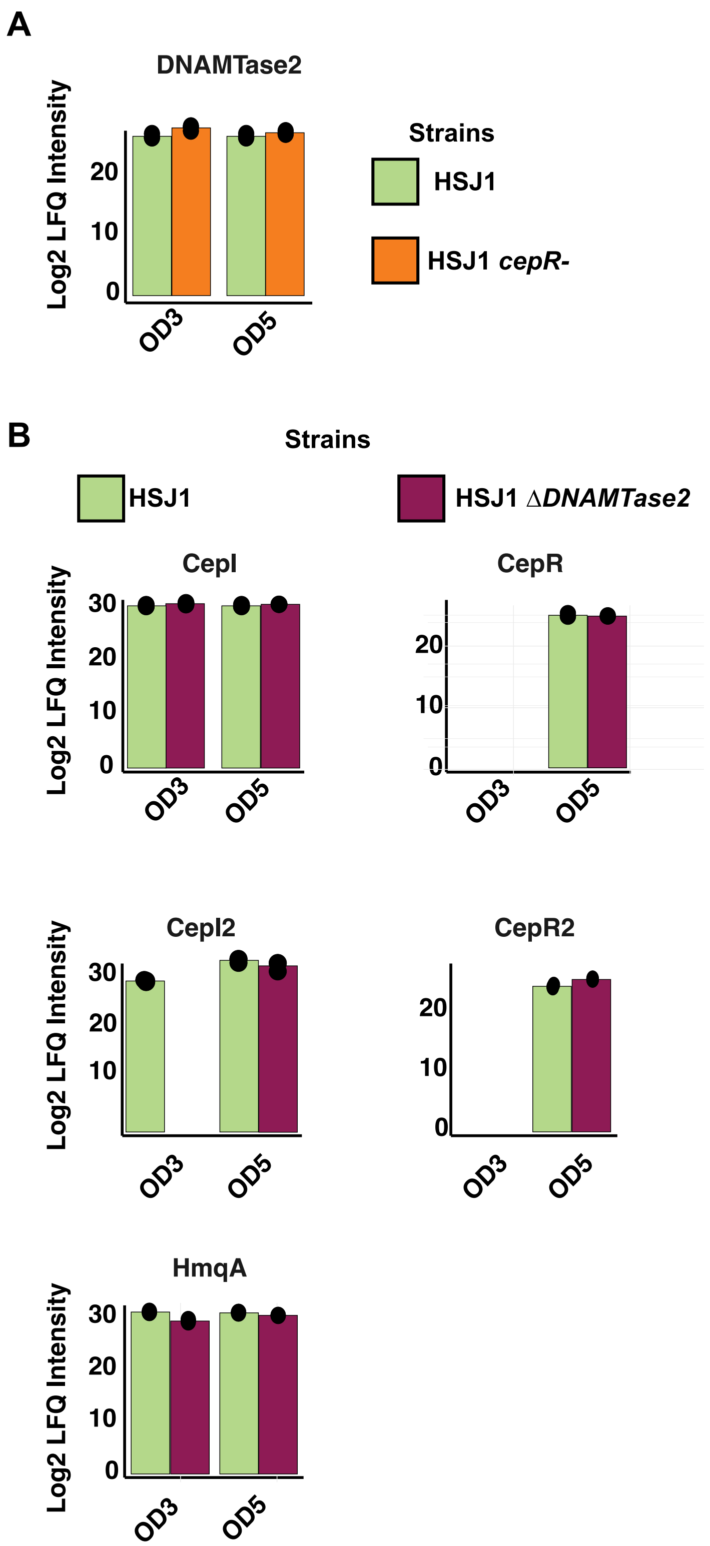

Figure S10. No interaction on protein levels between the Cep QS and the DNA MTase 2

A) Protein abundance of the DNA MTases in HSJ1, HSJ1v and HSJ1 *cepR-* QS mutant (Log_2 Fold change is represented on the graph). B) Protein or peptide abundance of the QS proteins in HSJ1, HSJ1v, HSJ1 Δ*DNAMTase 2* mutant (Log_2 Fold change is represented on the graph). Mean and replicates are shown on each figure.
